# Nonlinear Bayesian filtering and learning: a neuronal dynamics for perception

**DOI:** 10.1038/s41598-017-06519-y

**Published:** 2017-08-18

**Authors:** Anna Kutschireiter, Simone Carlo Surace, Henning Sprekeler, Jean-Pascal Pfister

**Affiliations:** 1 Institute of Neuroinformatics, University of Zurich/ETH Zurich, Zurich, Switzerland; 2 Neuroscience Center Zurich, University of Zurich/ETH Zurich, Zurich, Switzerland; 3 Department for Electrical Engineering & Computer Science, Technische Universität Berlin, Berlin, Germany; 4 Bernstein Center for Computational Neuroscience, Berlin, Germany

## Abstract

The robust estimation of dynamical hidden features, such as the position of prey, based on sensory inputs is one of the hallmarks of perception. This dynamical estimation can be rigorously formulated by nonlinear Bayesian filtering theory. Recent experimental and behavioral studies have shown that animals’ performance in many tasks is consistent with such a Bayesian statistical interpretation. However, it is presently unclear how a nonlinear Bayesian filter can be efficiently implemented in a network of neurons that satisfies some minimum constraints of biological plausibility. Here, we propose the Neural Particle Filter (NPF), a sampling-based nonlinear Bayesian filter, which does not rely on importance weights. We show that this filter can be interpreted as the neuronal dynamics of a recurrently connected rate-based neural network receiving feed-forward input from sensory neurons. Further, it captures properties of temporal and multi-sensory integration that are crucial for perception, and it allows for online parameter learning with a maximum likelihood approach. The NPF holds the promise to avoid the ‘curse of dimensionality’, and we demonstrate numerically its capability to outperform weighted particle filters in higher dimensions and when the number of particles is limited.

## Introduction

Since the seminal work of Helmholtz^1^, who hypothesized 150 years ago that perception can be seen as a process of unconscious inference, an increasing number of studies have shown that the brain performs perceptual tasks consistently with Bayesian inference^2,3^. In this perspective, perception relies on noisy and incomplete data that needs to be integrated across multiple sensory modalities and weighted according to sensory reliability. In addition, perception makes use of the strong statistical regularities of objects in our environment by forming prior beliefs about the world. Since our environment is fundamentally dynamic, the ability to adapt to changes in real time is essential for perception. This ‘Bayesian brain hypothesis’ is supported by ample experimental evidence, ranging from psychophysical findings^4–6^ to neuronal recordings^7–9^ that are in line with Bayesian computation. However, most of the studies concerned with the theory of perception consider fairly simple tasks, where the observations are generated either from static hidden variables^10^ or from hidden variables with a discrete state-space^11,12^, or the underlying dynamics are considered linear^13,14^.

In a dynamical setting, where temporally changing signals have to be estimated online from the history of observations, Bayesian inference is commonly referred to as ‘filtering’. In general, nonlinear Bayesian filtering is a challenging task even without the imperative of a plausible implementation on a neuronal architecture. If the prior distribution is a Gaussian and the noisy observations depend linearly on the hidden states, the inference problem is solved by the Kalman filter^15,16^, which has received substantial attention in the signal processing community and turns out to be of increasing importance in neuroscientific phenomenological modeling, e.g. in sensorimotor integration tasks^4^ or in estimating motor disturbances from an adaptive gain^17^. Solutions for most nonlinear, i.e. non-Gaussian, filtering problems^18,19^ are analytically intractable and thus have to be approximated.

On the algorithmic level, sampling-based approaches, which represent distributions with samples have proven to be a powerful tool to solve the nonlinear filtering problem numerically. In principle, they enable any posterior distribution to be represented with an accuracy that depends on the number of samples. On the one hand, so called particle filtering methods^20,21^ are well suited for dynamical priors, but suffer from inevitable weight decay over time, resulting in a ‘curse of dimensionality’ (COD) in higher-dimensional models. A widely used strategy to mitigate weight decay is particle resampling, but it can neither avoid the COD, nor does it increase implementability by a neuronal population. On the other hand, Langevin sampling^22,23^ and related techniques, such as the ‘fast sampler’^24^, provide a promising ground for a biologically plausible implementation of neural or synaptic sampling^25,26^, but are restricted to static generative models.

Following a sampling-based approach, we propose a framework for how the brain could perform filtering from noisy sensory stimuli. We formulate perception as the task of dynamical state estimation, which is set in the context of continuous-time continuous-state nonlinear filtering theory. Motivated by this theory, we propose a particle filter without importance weights, the Neural Particle Filter (NPF). The proposed filter can be implemented in a biologically realistic architecture with rate-based neuronal units. Specifically, the absence of importance weights allows an interpretation of this particle filter as a neuronal dynamics: task-specific neurons are identified with samples from the posterior (or ‘particles’). Consequently, this method does not suffer from weight-decay and the associated COD, both of which prohibit a biologically realistic implementation of weighted particle methods. For the parameter learning, we propose an online maximum likelihood approach. This approach leads to non-local interactions in the learning rules for synaptic connections. However, we find that Hebbian learning rules are recovered in the small-noise limit.

We show that the NPF algorithm exhibits properties that are considered essential for real-time perception: taking into account both observation noise and sensory ambiguities, it weighs prior knowledge and sensory information from different modalities to form an estimate of the real world hidden state, and it is able to adapt its internal model according to the observations. On the algorithmic assessment side, we show numerically that despite being technically a suboptimal filter, the NPF has a performance which is nearly indistinguishable from optimal filters in low dimensions. For higher dimensional problems, we provide numerical evidence that the NPF does not suffer from the COD and actually outperforms weighted particle filter methods when the number of particles is limited. The NPF thus can be seen as a biological implementation candidate of a computationally relevant filtering algorithm.

## Methods

### Nonlinear filtering as a generic computational task

We formulate the computational task in terms of the classical filtering problem with additive noise (for additional details, see refs^27,28^). The hidden state ${{\bf{x}}}_{t}\in {{\mathbb{R}}}^{n}$, which the brain cannot access directly (e.g. the position of prey), follows the Itô stochastic differential equation (SDE):

1 $$d{{\bf{x}}}_{t}={\bf{f}}({{\bf{x}}}_{t})\,dt+{{\rm{\Sigma }}}_{x}^{\mathrm{1/2}}\,d{{\bf{w}}}_{t},$$

with a nonlinear, deterministic drift function ${\bf{f}}({\bf{x}}):{{\mathbb{R}}}^{n}\to {{\mathbb{R}}}^{n}$ (for consistency, vectors will be printed in bold face). Stochastic diffusion is governed by the uncorrelated vector Brownian motion process ${{\bf{w}}}_{t}\in {{\mathbb{R}}}^{n}$ with noise covariance ${{\rm{\Sigma }}}_{x}\in {{\mathbb{R}}}^{n\times n}$.

At each moment in time, the hidden state **x**_*t*_ gives rise to noisy observations ${{\bf{y}}}_{t}\in {{\mathbb{R}}}^{m}$ that represent sensory input (e.g. visual or auditory input). The observation dynamics is again modeled in terms of an Itô diffusion, with a drift term following the hidden states via a generative function ${\bf{g}}({\bf{x}}):{{\mathbb{R}}}^{n}\to {{\mathbb{R}}}^{m}$ and a Brownian motion process **u**_*t*_, modulated by the sensory noise covariance ${{\rm{\Sigma }}}_{y}\in {{\mathbb{R}}}^{m\times m}$:

2 $$d{{\bf{y}}}_{t}={\bf{g}}({{\bf{x}}}_{t})\,dt+{{\rm{\Sigma }}}_{y}^{\mathrm{1/2}}\,d{{\bf{u}}}_{t}.$$

The functions **f** and **g** satisfy standard conditions frequently employed in nonlinear filtering (see for instance ref.^27^).

**Figure 1:**
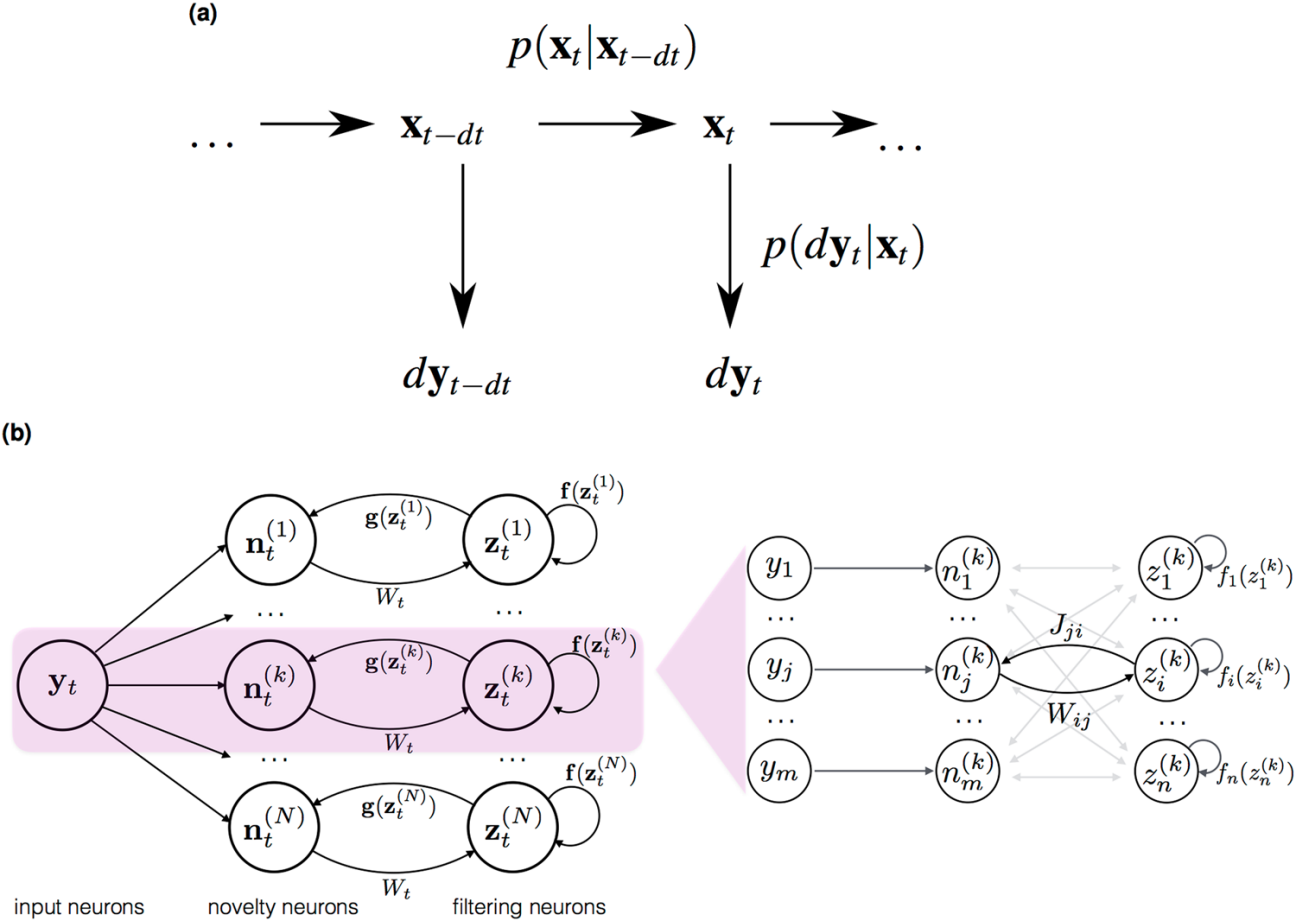
Generative model and neural network implementation. (**a**) Generative model defined by Eqs (1) and (2). (**b**) Implementation of Eq. (4) as a recurrent neuronal network. Left: each particle in the NPF corresponds to one out of *N* subnetworks, which run in parallel. Here, each circle denotes a neuronal population. Right: connections between neurons within the *k* ^th^ subnetwork in a model with a linear generative function ${\bf{g}}({\bf{x}})=J{\bf{x}}$. Novelty neurons *n*_*j*_ are connected to filtering neurons *z*_*i*_ via the decoding weight *W*_*ij*_. Feedback connections from filtering neurons *z*_*i*_ to novelty neurons *n*_*j*_ have the weight *J*_*ji*_. We further assume the self-interaction to be local, i.e. the components of the hidden dynamics are independent ${f}_{i}({{\bf{z}}}^{(k)})={f}_{i}({z}_{i}^{(k)})$. Here, each circle denotes a single neuron.

Together, Eqs (1) and (2) define a generative model (Fig. 1a). Specifically, Eq. (1) gives rise to a transition probability $p({{\bf{x}}}_{t}|{{\bf{x}}}_{t-dt})$ of the hidden state, and Eq. (2) gives rise to an emission probability (or observation likelihood) $p(d{{\bf{y}}}_{t}|{{\bf{x}}}_{t})$, respectively. Further, we assume the function **f**(**x**) is chosen such that a stationary probability density *p*(**x**_*t*_) of the hidden state exists (see SI, section 1.1), which serves as a prior over the hidden state.

Solving the filtering problem is the task of finding the posterior probability density $p({{\bf{x}}}_{t}|{{\mathscr{Y}}}_{t})$ of the hidden state, conditioned on the whole sequence of observations ${{\mathscr{Y}}}_{t}=\{{{\bf{y}}}_{s},s\in [0,t]\}$ up to time *t*. For a linear hidden drift function **f**(**x**) and a linear observation function **g**(**x**), this task is solved by the Kalman-Bucy filter^16^, which is a continuous-time version of the well-known Kalman filter. However, the solution to the *nonlinear* filtering problem is in general analytically intractable, because it suffers from the so-called closure problem (see SI, section S 1.2). Therefore, introducing a suitable approximation is an inevitable step when approaching the nonlinear filtering problem.

### Sampling-based representation

In the theoretical neuroscience literature, sampling-based approaches for filtering with a representation of the posterior as in Eq. (3) have not received much attention so far (one of the few examples can be found in refs^12,29^), although they have some experimental support^8,30^ and are considered relevant according to the ‘neural sampling hypothesis’^31^. Therefore, we would like to explore this approach further and hence approximate probability density functions in terms of a finite number of variables. For example, this can be achieved by taking *N* weighted samples:

3 $$p({\bf{x}},t)\approx \sum _{k=1}^{N}\,{w}_{t}^{(k)}\,\delta ({\bf{x}}-{{\bf{z}}}_{t}^{(k)}),\,{\rm{with}}\,\sum _{k=1}^{N}\,{w}_{t}^{(k)}=1.$$

Thus, the probability of the random variable to have a certain value range is proportional to the relative number of samples within this range, weighted by their respective weight ${w}_{t}^{(k)}$.

Filtering algorithms representing the posterior in this sampling-based manner are commonly referred to as particle filters (PF). In standard PFs, update rules for the sample (or ‘particle’) trajectories ${{\bf{z}}}_{t}^{(k)}$, as well as the weights ${w}_{t}^{(k)}$ are given^32^. Despite asymptotic convergence to the true posterior for an infinite number of particles, this approach has two disadvantages: first, one finds numerically that after a finite number of time-steps most particle weights decay to zero, which depletes the number of effective samples. Weight decay is an undesirable trait of weighted particle methods in general. Second, the problem is exacerbated if the number of dimensions of the hidden state **x**_*t*_ is large. In this case, the number of particles needed for good numerical performance grows exponentially with the number of dimension, a variant of the COD^33^. The first problem is usually resolved by resampling, and the second problem has been addressed e.g. in ref.^34^, but it is an open question how these purely numerical strategies can be performed by neuronal units. To bypass this question, we consider a particle filter with equally weighted samples, i.e. ${w}^{(k)}=1/N$ for all *k*.

### Filtering with the Neural Particle Filter

As an inference algorithm, we propose an SDE that governs the dynamics of particles ${{\bf{z}}}_{t}^{(k)}$. Let us consider *N* i.i.d. stochastic processes ${{\bf{z}}}_{t}^{(k)}$, $k=1,\ldots ,N$, conditioned on the observations ${{\mathscr{Y}}}_{t}$, following the Itô diffusion

4 $$d{{\bf{z}}}_{t}^{(k)}={\bf{f}}({{\bf{z}}}_{t}^{(k)})\,dt+{W}_{t}(d{{\bf{y}}}_{t}-{\bf{g}}({{\bf{z}}}_{t}^{(k)})\,dt)+{{\rm{\Sigma }}}_{x}^{1/2}\,d{{\boldsymbol{\omega }}}_{t},$$

where ${{\boldsymbol{\omega }}}_{t}\in {{\mathbb{R}}}^{n}$ is an uncorrelated vector Brownian motion process and ${W}_{t}\in {{\mathbb{R}}}^{n\times m}$ is a time-dependent gain matrix or decoding weight matrix.

Equation (4), which we will further refer to as the Neural Particle Filter (NPF), is an ansatz that serves as a sampling-based approximation to the nonlinear filtering problem: each of the *N* stochastic processes ${{\bf{z}}}_{t}^{(k)}$ is a conditionally independent empirical sample, or particle, from the distribution $p({{\bf{x}}}_{t})=1/N{\sum }_{k=1}^{N}\,\delta ({{\bf{x}}}_{t}-{{\bf{z}}}_{t}^{(k)})$, which approximates the true posterior $p({{\bf{x}}}_{t}|{{\mathscr{Y}}}_{t})$ at every time *t*, i.e. $p({{\bf{x}}}_{t}|{{\mathscr{Y}}}_{t})\approx 1/N{\sum }_{k=1}^{N}\,\delta ({{\bf{x}}}_{t}-{{\bf{z}}}_{t}^{(k)\,})$. Expectations with respect to the posterior 〈·〉 are approximated according to

5 $${\mathbb{E}}[\phi ({{\bf{x}}}_{t})|{{\mathscr{Y}}}_{t}]=\langle \phi ({{\bf{x}}}_{t})\rangle \approx \frac{1}{N}\sum _{k}\,\phi ({{\bf{z}}}_{t}^{(k)}).$$

The ansatz in Eq. (4) is motivated by the formal solution to the filtering problem, more precisely by the dynamics of the first posterior moment (Eq. S-16 in SI) and shares some important properties with classical filtering methods: first, it is governed by both the dynamics of the hidden process **x**_*t*_ and by a correction proportional to the so-called innovation term $d{{{\bf{n}}}^{(k)}}_{t}=d{{\bf{y}}}_{t}-{\bf{g}}({{\bf{z}}}_{t}^{(k)})\,dt$. The innovation term compares the sensory input *d* **y**_*t*_ with the current prediction ${\bf{g}}({{\bf{z}}}_{t}^{(k)})\,dt$ according to the single particle position, and thus can be seen as a predictive error signal^10^. Second, the gain matrix *W*_*t*_ determines the emphasis that is laid on new information via observations *d* **y**_*t*_. This is conceptually similar to the Kalman gain^15,16^ for a linear model.

The gain *W*_*t*_ can, for instance, be computed according to ${W}_{t}={\rm{cov}}({{\bf{x}}}_{t},{\bf{g}}{({{\bf{x}}}_{t})}^{T})\,{{\rm{\Sigma }}}_{y}^{-1}$, an empirical choice motivated by Eq. S-16. This gain adjusts according to the observation noise ${{\rm{\Sigma }}}_{y}$ as well as to the spatial ambiguity as measured by the covariance between the state **x**_*t*_ and the generative function **g**(**x**_*t*_). This covariance cannot be accessed directly, but is estimated empirically and instantaneously from the particle positions via Eq. (5) (cf. also Eq. 6). The resulting filtering algorithm with empirically determined gain is summarized in algorithm 1.

The gain introduces a weighting between the prior probability distribution *p*(**x**_*t*_) induced by Eq. (1), and the likelihood function *p*(*d* **y**_*t*_|**x**_*t*_) induced by Eq. (2) and thus serves as a measure for the peakedness of the likelihood. If the observation noise is small, the decoding weight is large. Then, the dynamics in Eq. (4) will entirely be determined by the innovation term, and the inter-particle variability governed by the diffusion term will be negligible. In this limit, the deterministic observation limit, a single sample from Eq. (4) suffices to represent the posterior. On the other hand, if the decoding weight is zero, new information is disregarded, and each sample evolves just like an i.i.d. copy of Eq. (1). In this case, the resulting posterior density simply equals the stationary prior density *p*(**x**_*t*_). Further details on these limits can be found in SI, section S 2.1.

### Algorithm 1

The **Neural Particle Filter algorithm** with empirical gain function. The NPF continuously extracts hidden features ***z***_*t*_ from an input stream of observations ***y***_*t*_. The distribution of features is represented by *N* populations of neurons.

1: **procedure** NPF $$({\{{{\bf{z}}}_{t-\delta t}^{(k)}\}}_{k=1}^{N},\delta {{\bf{y}}}_{t})$$

2:Computes the weight matrix that connects novelty neurons to filter neurons

6 $${W}_{t}=\frac{1}{N}\sum _{k=1}^{N}{{\bf{z}}}_{t-\delta t}^{(k)}{\bf{g}}{({{\bf{z}}}_{t-\delta t}^{(k)})}^{T}-\frac{1}{{N}^{2}}\sum _{k,l=1}^{N}{{\bf{z}}}_{t-\delta t}^{(k)}{\bf{g}}{({{\bf{z}}}_{t-\delta t}^{(l)})}^{T}$$

3:**for** *k* = 1 to *N* **do**

4: update novelty neurons **n** ^(*k*)^

7 $$\delta {{\bf{n}}}_{t}^{(k)}=\delta {{\bf{y}}}_{t}-{\bf{g}}({{\bf{z}}}_{t-\delta t}^{(k)})\delta t$$

5: update filter neurons **z** ^(*k*)^

8 $${{\bf{z}}}_{t}^{(k)}={{\bf{z}}}_{t-\delta t}^{(k)}+{\bf{f}}({{\bf{z}}}_{t-\delta t}^{(k)})\delta t+{W}_{t}\delta {{\bf{n}}}_{t}^{(k)}+\sum _{x}^{1/2}\delta {\omega }_{t}^{(k)}\quad {\rm{with}}\quad \delta {\omega }_{t}^{(k)} \sim {\mathscr{N}}(0,\delta t)$$

**6:end for**

**7:return** $${\{{{\bf{z}}}_{t}^{(k)}\}}_{k-1}^{N}$$

**8: end procedure**

### Parameter learning

In a more general setting, model parameters *θ* of Eqs (1) and (2) may not or only partially be known, and thus need to be learned online from the stream of observations ${{\mathscr{Y}}}_{t}$. In this case, the NPF algorithm can be extended to include a parameter update that performs an online gradient ascent on the log likelihood of the whole history of observations ${{\mathscr{Y}}}_{t}$:

9 $${L}_{t}(\theta )={\int }_{0}^{t}\,{\langle {{\bf{g}}}_{\theta }({{\bf{x}}}_{s})\rangle }_{\theta }^{T}\,{{\rm{\Sigma }}}_{y}^{-1}\,d{{\bf{y}}}_{s}-\frac{1}{2}{\langle {{\bf{g}}}_{\theta }({{\bf{x}}}_{s})\rangle }_{\theta }^{T}\,{{\rm{\Sigma }}}_{y}^{-1}\,{\langle {{\bf{g}}}_{\theta }({{\bf{x}}}_{s})\rangle }_{\theta }\,ds,$$

which in turn is computed directly from the approximated filtering distribution itself via empirical estimates (Eq. 5). Note the dependence of the conditional estimate on the model parameters *θ*, which has to be taken into account when attempting to maximize the log likelihood. It can be shown that maximizing this log likelihood is equivalent to minimizing the prediction error in continuous time (cf. SI, section 3.1.2).

Model parameters *θ* are learned by online gradient ascent on the log likelihood, giving rise to the following learning rules for the parameters ﻿*θ*:

10 $${\eta }_{\theta }^{-1}d\theta ={(\frac{{\rm{\partial }}}{{\rm{\partial }}\theta }\langle {{\bf{g}}}_{\theta }({{\bf{x}}}_{t}){\rangle }_{\theta })}^{T}\,{{\rm{\Sigma }}}_{y}^{-1}(d{{\bf{y}}}_{t}-\langle {{\bf{g}}}_{\theta }({{\bf{x}}}_{t}){\rangle }_{\theta }\,dt).$$

This online learning approximation is justified if the time scale of learning is much larger than the dynamics of the filter, i.e. for small learning rates.

In Eq. (10) the novelty signal $d{{\bf{y}}}_{t}-\langle {\bf{g}}({{\bf{x}}}_{t})\rangle \,dt$ is multiplied with a parameter gradient on the posterior estimate of the generative function $\langle {\bf{g}}({{\bf{x}}}_{t})\rangle$. Thus, we have to take into account the implicit change of the posterior distribution with respect to the model parameters, the so called ﻿*filter derivative*﻿. The problems of tractability of the filter also apply to filter derivatives, and the latter have to be approximated with particle representations (see SI sections S 3.1 and S 3.2, particularly algorithm S-1 for further details and for derivation of likelihood and learning rules, following ref.^35^).

The learning rules for the parameters in Eq. (10) are non-local, i.e. they depend on aggregated states of the entire set of particles. In the limit of small observation noise and for a linear observation model ${\bf{g}}({\bf{x}})=J{\bf{x}}$, the learning rule for the generative matrix *J* (or mixing matrix) can be approximated by a local and Hebbian learning rule:

11 $$dJ\propto (d{{\bf{y}}}_{t}-J\langle {{\bf{x}}}_{t}\rangle \,dt)\,{\langle {{\bf{x}}}_{t}\rangle }^{T}\approx \langle (d{{\bf{y}}}_{t}-J{{\bf{x}}}_{t}){{\bf{x}}}_{t}^{T}\rangle ,$$

It is important to mention that not only the model parameters in Eqs (1) and (2), but also the decoding parameters, i.e. components of the gain matrix *W*_*t*_, can be learned with a maximum likelihood approach, as opposed to setting the gain according to the empirical estimate from the particle positions. The learning rules for the components of the gain matrix read:

12 $$d{W}_{ij}={\eta }_{W}{(\frac{\partial }{{\partial }_{{W}_{ij}}}\langle {\bf{g}}({{\bf{x}}}_{t})\rangle )}^{T}\,{{\rm{\Sigma }}}_{y}^{-1}(d{{\bf{y}}}_{t}-\langle {\bf{g}}({{\bf{x}}}_{t})\rangle dt),$$

This alternative to determining the gain corrects for the heuristic ansatz of the NPF equation (4) by determining the decoding weights rigorously. In fact, it can be shown that parameter learning with a maximum likelihood approach is able to make up even for a very poor filtering ansatz by setting parameters accordingly^36^.

### Neuronal Implementation

The NPF can be interpreted in terms of a neuronal dynamics and the algorithm can be implemented in a recurrent neuronal network. Specifically, we consider the dynamics of a population of *N* × *n* filter neurons **z** ^(*k*)^, whose analog neuronal activities (for instance instantaneous firing rate) represent samples of the posterior, in line with the neural sampling hypothesis^31^ (Fig. 1b). The computation is performed in *N* subnetworks, one for each ‘particle’ in the NPF. The architecture of each parallel subnetwork is structurally similar to the one introduced in Rao & Ballard^10^ (compare Fig. 1A in this reference). As input to the subnetworks we consider a neuronal population **y**_*t*_ whose rates are evoked from the underlying hidden stimulus **x**_*t*_ via the generative dynamics in Eq. (2).

Within each subnetwork *k* there are two types of neurons, novelty neurons **n** ^(*k*)^ and filtering neurons **z** ^(*k*)^. The population of novelty neurons **n** ^(*k*)^ receives input from the population of sensory neurons **y** and are recurrently connected to the population of filtering neurons **z** ^(*k*)^ (see Eq. 7). The output of novelty neuron ${{\bf{n}}}_{t}^{(k)}$ represents the residual between the actual sensory input and the expected input within this subnetwork (single-particle prediction error). The output of the novelty neurons is received by the filtering neurons **z** ^(*k*)^ via the feedforward synaptic weights *W*_*t*_ (see Eq. 8). Therefore, the dynamics of filter neurons depends on both sensory inputs (via the novelty neurons) and on prior dynamics (via the nonlinear function ${\bf{f}}({{\bf{z}}}_{t}^{(k)})$). The output of the filtering neurons in each subnetwork corresponds to a single particle state in the particle filter.

In this implementation, *W*_*t*_ corresponds to the matrix of synaptic weights that connects novelty neurons **n** ^(*k*)^ to filtering neurons **z** ^(*k*)^. If the generative function ${\bf{g}}({{\bf{x}}}_{t})=J{{\bf{x}}}_{t}$ is linear, then *J* denotes the matrix of feedback weights which connects filtering neurons to novelty neurons (Fig. 1b, right). In general, the learning rules for these weights, which arise from maximizing the likelihood in Eq. (9), are not local, i.e. they rely on the state of the whole network (Eq. 10, cf. SI section S 3.1). However, in the deterministic limit the learning rule for the generative weight matrix *J* can be replaced by a learning rule that is both Hebbian and local and relies on a multiplication between pre- and postsynaptic activity (Eq. 11), i.e. between filtering and novelty neurons. Further, for small observation noise, *W*_*t*_ can be replaced by a constant matrix without affecting the filtering performance (as long as the weights are large compared to the prior dynamics). Therefore, at least in this limit, the network presented in Fig. 1b is implementable as a neuronal dynamics of a recurrent network with local Hebbian synaptic plasticity.

## Results

Following a top-down approach, we are investigating how the brain can implement dynamic perception. First, we interpret perception as the computational task of nonlinear Bayesian filtering. The solution to the nonlinear filtering problem is in general infinite-dimensional, and thus needs a finite-dimensional approximation. By choosing to represent the time-varying posterior in terms of empirical samples, which are propagated according to the NPF equation (4), we can formulate such a finite-dimensional approximation. Further, the samples (or ‘particles’) are directly identified with the activity of filtering neurons, and the NPF equation with their neuronal dynamics. Thus, we are able to base the implementation of the algorithm on a neuronal architecture.

With a simple example, we are going to illustrate that our algorithm captures the following key properties of perception^3^: (1) it relies on noisy and incomplete sensory data, (2) it uses prior knowledge of the dynamic structure of the environment, (3) it efficiently combines information from several sensory modalities, and (4) it can dynamically adapt to changes in the environment.

### Perception as nonlinear filtering

**Figure 2:**
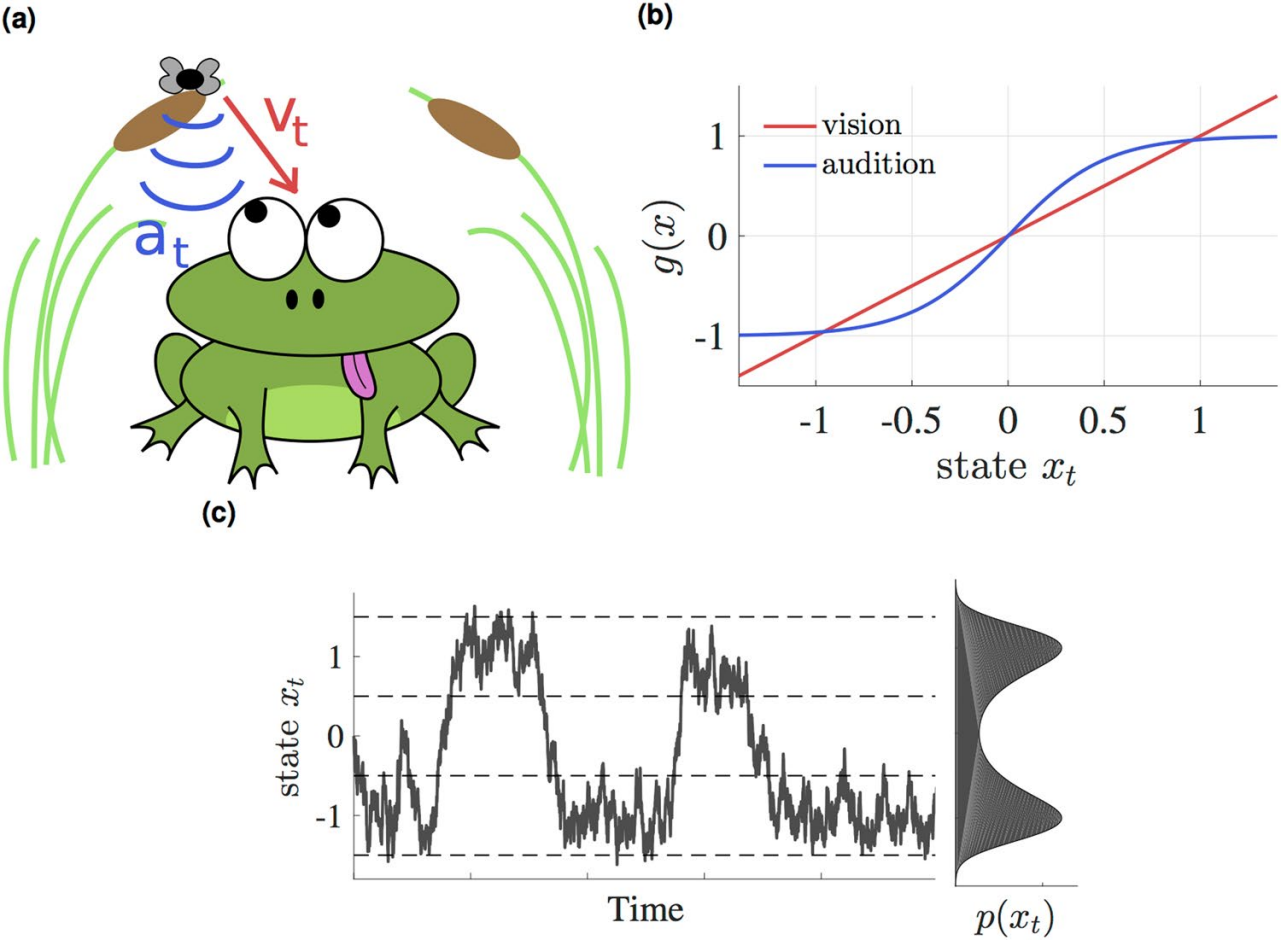
A toy model illustrating the filtering task for perception. (**a**) Cartoon of a frog tracking a fly, relying on its visual (*v*_*t*_) and auditory (*a*_*t*_) channels. (**b**) Nonlinearities in the generative function *g*(*x*) of the two sensory channels. Vision is modeled as a linear mapping, while audition is modeled as a sigmoid function. (**c**) A sample trajectory of the fly according to Eq. (15). Note that the nonlinearity in the drift gives rise to a bimodal stationary distribution *p*(*x*_*t*_).

Consider a frog who sits below two branches and is tracking an insect flying between two branches (Fig. 2a). The frog cannot directly observe the position *x*_*t*_ of the insect, which will further be referred to as the ‘hidden state’, but instead has to rely on two sensory channels, a visual (*v*_*t*_) and an auditory (*a*_*t*_) channel. These observations are evoked by the hidden state via generative functions (Fig. 2b), which enter as a deterministic drift in the dynamics of the observations:

13 $$d{v}_{t}={x}_{t}\,dt+{\sigma }_{v}\,d{\beta }_{t},$$

14 $$d{a}_{t}=\,\tanh (2{x}_{t})\,dt+{\sigma }_{a}d{\gamma }_{t}.$$

*β*_*t*_ and *γ*_*t*_ are independent Brownian motion processes that model sensory noise, making *v*_*t*_ and *a*_*t*_ conditionally independent. The nonlinearity in the auditory channel (Eq. 14) is motivated by the fact that sound localization depends on interaural difference^37^, which we model as a sigmoid in this 1D example.

In addition, the frog has some prior knowledge about the dynamics of the fly, which we model as

15 $$d{x}_{t}=3{x}_{t}\mathrm{(1}-{x}_{t}^{2})\,dt+d{w}_{t},$$

where the Brownian motion process *w*_*t*_ accounts for noise due to the erratic behavior of the insect. A sample trajectory from this stochastic process is shown in Fig. 2c. Note that the nonlinearity of the drift function in this dynamics gives rise to a bimodal stationary distribution for the position of the insect.

In order to track the fly, the frog has to integrate the information from its sensory input and combine it with its prior knowledge in order to compute the posterior density $p({x}_{t}|{V}_{t},{A}_{t})$, i.e. the probability to find the fly in a certain spatial region given the visual and auditory sensory streams ${V}_{t}=\{{v}_{s};0\le s\le t\}$ and ${A}_{t}=\{{a}_{s}\mathrm{;0}\le s\le t\}$. This task is commonly referred to as nonlinear filtering. Due to the nonlinear dynamics of the hidden and observation processes, the solution to this particular example is analytically intractable and thus requires an approximation.

**Figure 3:**
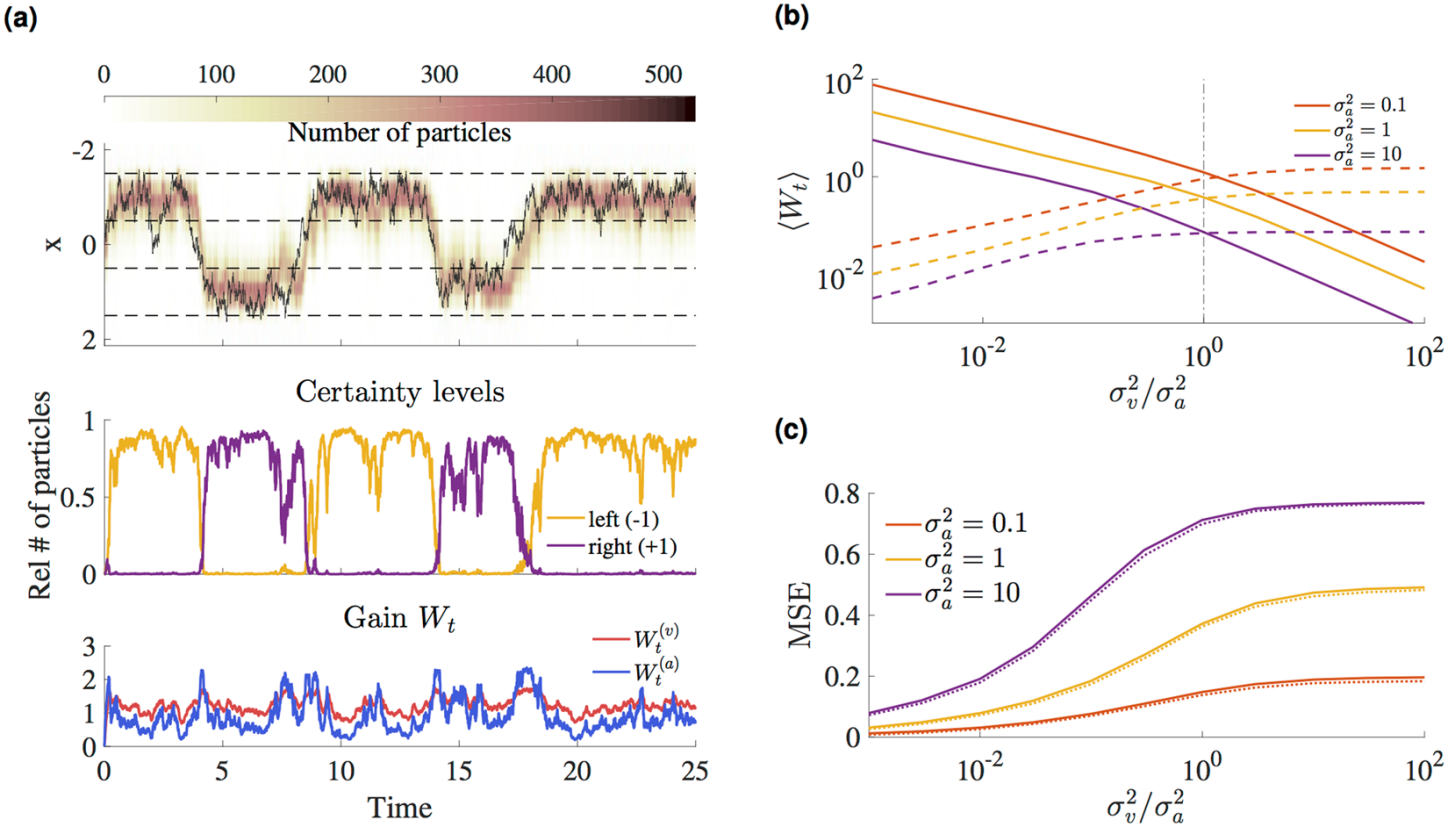
The NPF as a model for perception for multisensory perception. (**a**) Tracking simulation with *N* = 1000 filtering neurons and sensory noise ${\sigma }_{v}^{2}={\sigma }_{a}^{2}=0.1$. The upper panel shows the true trajectory of the insect (solid black line) and particle densities. The regions between the dotted black lines denote the two branches, and certainty levels in the middle panel correspond to the relative number of particles whose states are within one of the two branches. At each time, the sensory gains in the lower panel are computed according to Eqs (17) and (18). (**b**) Time-averaged gains ${\langle {W}_{t}^{(v)}\rangle }_{t}$ (solid line) and ${\langle {W}_{t}^{(a)}\rangle }_{t}$ (dashed line) as function of sensory noise in multisensory integration. (**c**) Performance in terms of time-averaged MSE (dotted lines: PF) using both sensory cues *v*_*t*_ and *a*_*t*_.

We propose that this task is solved by a set of *N* filtering neurons ${z}_{t}^{(k)}$, $k=1,\ldots ,N$. The empirical distribution of neuronal activities ${z}_{t}^{(k)}$ approximately samples the posterior density, thereby acting as a weight-less particle filter that successfully tracks the position of the insect (Fig. 3a). The state estimate ${\hat{x}}_{t}$ (posterior mean) can be read out from this population by averaging the activities of the filtering neurons, i.e. ${\hat{x}}_{t}\approx \langle {z}_{t}\rangle ={N}^{-1}{\sum }_{k}\,{z}_{t}^{(k)}$.

The neuronal dynamics of these filtering neurons are given by the NPF (Eq. 4) and for this particular example read:

16 $$\begin{array}{ccc}d{z}_{t}^{(k)} & = & 3{z}_{t}^{(k)}(1-{({z}_{t}^{(k)})}^{2})\,dt+d{\omega }_{t}^{(k)}\\  &  & +{W}_{t}^{(v)}(d{v}_{t}-{z}_{t}^{(k)}\,dt)+{W}_{t}^{(a)}(d{a}_{t}-\tanh (2{z}_{t}^{(k)})\,dt).\end{array}$$

Firstly, this dynamics is governed by the dynamics of the fly (Eq. 15), which serves as a prediction according to the frog’s prior knowledge about the position of the fly. Second, the prediction is corrected by the novelty of the observations in the sensory channels. The influence of the novelty is modulated by the two components of the gain matrix ${W}_{t}^{(v)}$ and ${W}_{t}^{(a)}$. A possible network implementation of this dynamics is depicted in Fig. 1b.

In approximately solving a filtering task, our model readily captures the first two key properties of perception, i.e. it relies on noisy and ambiguous observational data and is modified by prior knowledge of the dynamics of the fly.

The potential of having a description of the *full* posterior stretches far beyond simple state estimation, where one is only interested in the first moment. Particularly the sampling-based approximation of this posterior allows a convenient estimation of other relevant quantities. For example, the frog might want to know on which branch the insect is sitting in order to catch it more easily. It could directly deduce a certainty level for the left and right branch, respectively (Fig. 3a), by counting the number of samples within a certain activity range.

#### Cue integration

The decoding weights, or gain factors, ${W}_{t}^{(v)}$ and ${W}_{t}^{(a)}$, are essential for multisensory integration. They balance the relative effects of the two sensory modalities and the prior on the dynamics of the filtering neurons and thus quantify the reliability of the sensory channels. Here, we consider the empirically estimated gain ${W}_{t}={\rm{cov}}({{\bf{x}}}_{t},{\bf{g}}({{\bf{x}}}_{t}))\,{{\rm{\Sigma }}}_{y}^{-1}$ in the NPF. In this example, the weights evaluate to

17 $${W}_{t}^{(v)}={\rm{v}}{\rm{a}}{\rm{r}}({x}_{t})\,{\sigma }_{v}^{-2},$$

18 $${W}_{t}^{(a)}={\rm{c}}{\rm{o}}{\rm{v}}({x}_{t},\tanh (2{x}_{t})){\sigma }_{a}^{-2}.$$

The gains adjust according to sensory noise levels, i.e. the gains decrease on average for increasing noise level within one channel (Fig. 3b). In addition, they adjust according to ‘spatial’ ambiguity evoked by the nonlinearity in the observation function. More precisely, the gains are governed by the covariance between the state and the generative function, which is related to the slope of the nonlinearity. Loosely speaking, the more the generative function *g*(*x*_*t*_) changes with respect to the (currently estimated) position *x*_*t*_, the more reliable the observation. In this example, the sigmoid observation function of the auditory channel *a*_*t*_ is more reliable if the fly is considered to be in the center, and thus the auditory gain adjusts accordingly (lower panel of Fig. 3a).

Thus, the gains become large if a channel is particularly reliable, and in extreme cases dominate the dynamics of the filtering neurons, corresponding to the deterministic observation limit. The appropriate weighting of sensory information allows the neurons to solve the filtering task near-optimally and comparable to a standard PF, which is for example reflected by our simulation results in Fig. 3c (see also Fig. S-1 in SI).

#### Adaption of internal model

In our example, the frog could successfully track the position of the insect, but it could only do so because it had access to the generative model parameters in its internal model, i.e. it knew the prior dynamics of the insect and it was aware of how the sensory percepts were generated from the true state of the insect. Also, knowledge of these model parameters were crucial for determining the sensory weights ${W}_{t}^{(v)}$ and ${W}_{t}^{(a)}$ and thus significantly influenced the dynamics of the filtering neurons. However, the external world, represented by the model parameters, changes over time, and successful perception should adapt the internal model accordingly.

**Figure 4:**
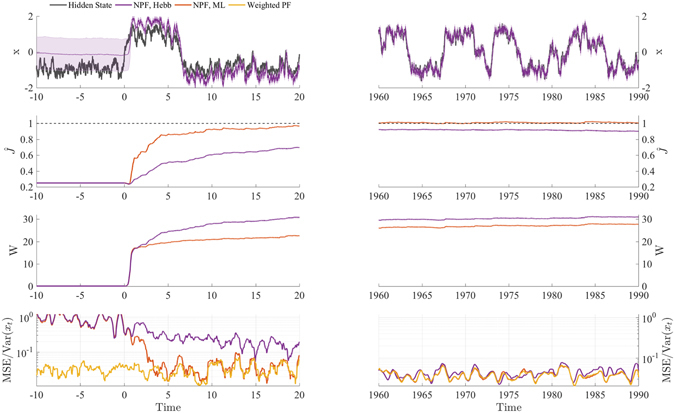
Model parameters are learned by a stochastic gradient ascent on the log likelihood. Simulations shown here correspond to the example model with only a visual cue, i.e. a generative model with Eqs (13) and (15). The generative weight *J* is learned online either by maximum likelihood (ML, Eq. 10) or the Hebbian learning rule (Hebb) in Eq. (11), which is a valid approximation for small sensory noise. The sensory gain *W*_*t*_ is learned online simultaneously, using Eq. (12). As benchmark, we use a weighted PF with the true model parameters. For both parameters *W*_*t*_ and *J*, learning starts at *t* = 0. As $\hat{J}$ approaches the true value *J* = 1, the trajectory of the filtering neurons (purple) is able to follow that of the true hidden state (black), and the MSE of the NPF resembles that of the standard PF.

We illustrate the learning of generative model parameters using our example with maximum likelihood (ML, Eq. 10). Further, we consider the limit of small sensory noise, which leads to Hebbian learning in the neuronal network (Fig. 1b, cf. Eq. 11). This time, the frog only relies on his visual channel *v*_*t*_, but in addition to tracking the insect, it also has to learn the generative factor *J* in the function $g(x)=Jx$, which relates the position of the insect to the visual input. Simultaneously, it also learns the gain ${W}_{t}^{(v)}$ according to Eq. (12) and with that implicitly an estimate of the reliability of its visual input. Figure 4 shows that this identification problem can be solved efficiently by the NPF, with an MSE that gradually approaches that of the benchmark (a standard PF with the ground-truth parameters) as the estimate of the parameters gets more accurate (see also section S 3.3).

### Algorithmic assessment

**Figure 5:**
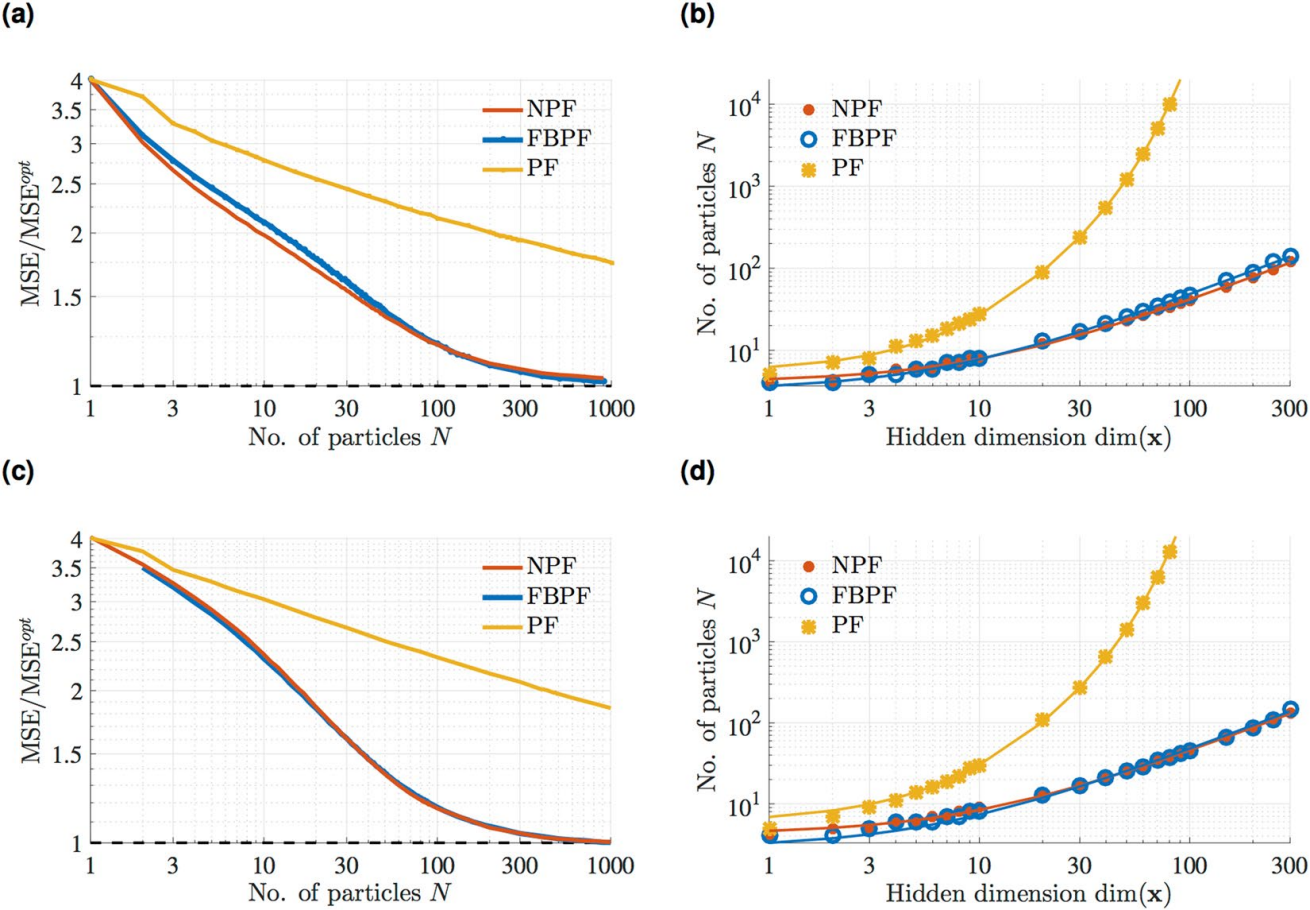
The NPF avoids the ‘curse of dimensionality’. (**a**) Filtering performance in terms of MSE (normalized to optimal performance, in this case $MS{E}^{{\rm{o}}{\rm{p}}{\rm{t}}}=0.5d$, whe﻿re *d* denotes the number of hidden dimensions) for varying number of particles for a linear model with high-dimensional hidden state-space (*d* = 80). Both unweighted approaches NPF and FBPF outperform the (standard) weighted particle filter for a limited number of particles. (**b**) Number of particles needed to achieve a numerical performance $MSE < 1.5MS{E}_{opt}$. The number of particles *N* needed for a standard (weighted) PF grows exponentially with *d* due to fast weight decay in higher dimensions. Contrarily, the unweighted approaches avoid the COD and the number of particles scales linearly with hidden dimensions. Solid lines correspond to linear ($N(d)=a\cdot d+b$, NPF: *a* = 0.38, *b* = 4.1 FBPF: *a* =0.45, *b* = 3.2) and exponential ($N(d)={c}_{0}\cdot {e}^{{c}_{1}d}+{c}_{2}\cdot d+{c}_{3}$ PF: *c*_0_ = 47, *c*_1_ = 0.07, *c*_2_ = -2.4, *c*_3_ = -42) least-squares fits. (**c**,**d**) Same as (**a**,**b**), but for nonlinear hidden dynamics with a bimodal stationary distribution. Least-squares fit as coefficients: *a* = 0.42, *b* = 4.2 (NPF), *a* = 0.45, *b* = 2.8 (FBPF), *c*_0_ = 44, *c*_1_ = 0.07, *c*_2_ = -2.1, *c*_3_ = -38 (PF).

The NPF equation (Eq. 4) is structurally very similar to a filtering algorithm called the Feedback Particle Filter^38,39^ (FBPF, see section S 1.3.2 in SI). The main difference between the NPF outlined in algorithm 1﻿ and the FBPF with a constant-gain approximation (used to make the computation of gain feasible in higher dimensions^39^) is a different prediction of the observation used in the innovation term, such that the NPF is consistent with a network implementation as shown in Fig. 1b. This important difference, however, does not negatively impact the performance of the NPF compared to that of the FBPF, as we demonstrate in Fig. 5 for a linear model.

For a weightless particle-filtering approach such as the NPF (or the FBPF), we can confidently say that the scaling of the required number of particles with dimension is less than exponential (Fig. 5) and thus avoids the COD in our example simulations. We find that the NPF using ${W}_{t}={\rm{cov}}({{\bf{x}}}_{t},{\bf{g}}({{\bf{x}}}_{t}))\,{{\rm{\Sigma }}}_{y}^{-1}$ performs well even for a limited number of samples, and the number of particles needed for a certain performance grows linearly with an increase in hidden dimensions. The standard PF, however, exhibits an exponential scaling in the number of particles, illustrating the COD.

In other words, despite the NPF being a sub-optimal filtering algorithm, just a limited number of particles suffices to solve the filtering task in higher dimensions with an acceptable performance. This robustness in performance for smaller number of particles is mainly due to the direct influence of the observations *d* **y**_*t*_ on the trajectories of the samples. In the unweighted approaches we show here (NPF and FBPF), each particle state itself can be seen as a point estimate of the state, which becomes exact for very small observation noise ${{\rm{\Sigma }}}_{y}$. Of course, the larger ${{\rm{\Sigma }}}_{y}$ becomes, the less the true posterior resembles a *δ*-function and the more particles are needed to account for its shape in general.

Besides being just an algorithmic trait, the scaling with dimensions does have biological relevance. Consider for instance our example from the previous subsection: thus far, the hidden state (the position of the insect) was considered purely one-dimensional. In a more realistic setting, the brain faces a much larger number of hidden states it has to infer, ranging from the position of an object in three-dimensional space to the relative presence of features making up a visual scene. Therefore, any filtering algorithm employed by a neuronal population for perception should be economical in its resources: an algorithm that needs an exponential amount of filtering neurons with growing dimension, i.e. an algorithm suffering from a COD, would be devastating. Instead, the number of neurons needed to solve the filtering task to a certain performance level should scale well with the number of hidden variables, a requirement that is fulfilled by a weightless particle-filtering approach such as the NPF.

## Discussion

In this paper, we set perception in the context of the computational task of nonlinear Bayesian filtering. Motivated by the theory of nonlinear filtering, we proposed an analog dynamics for particles (or neurons) that serves as a weight-free particle filter, the NPF. The NPF both inherently reflects important properties considered crucial for perception, and is further implementable as a neuronal dynamics in a recurrent neuronal network. It may thus serve as a step towards understanding how perception can be implemented in the brain on a conceptual level.

The NPF equation we propose in Eq. (4) is particularly suited to model perception phenomenologically, because it shares some important properties with perception. First, perception relies on noisy and often ambiguous and incomplete sensory data, as for instance encountered in visual scenes, and uses these to make sense of the world, which in our model is reflected by inferring the hidden state variable. Second, the brain needs to combine different sensory cues efficiently in order to decrease uncertainty or ambiguity. In addition, it exploits strong statistical regularities of the environment by taking into account prior knowledge. In the NPF, multi-sensory integration is realized as a weighted sum of sensory input, where the weights of the modalities balance their respective significance. In particular, the weight of a single modality adjusts according to its reliability, i.e. it decreases with increasing ambiguity or sensory noise level. This has also been reported experimentally^6^. Because prior dynamics directly enter the NPF, prior knowledge about the environment is automatically incorporated and can in principle be learned. Lastly, perception should be able to adapt to changes in the environment, which is taken into account by a dynamical gain and online parameter updates.

There are two main competing proposals about how probability distributions underlying Bayesian computations might be represented in the brain. Firstly, it has been suggested that probability distributions are expressed as probabilistic population codes^40^ (PPC), in which each neuron represents a state of the encoded random variable and their activities are proportional to the (log) probability of the corresponding state. Filtering approaches based on population codes have been explored in the literature for a large set of models^13,14,41,42^. In this representation, neurons directly correspond to the parameters of the distribution, and thus the critical factor for accuracy is the number of neurons. Further, they all suffer from the COD for multimodal distributions.

The second proposal, called neural sampling hypothesis^31^, uses an inference scheme where the activity of each neuron represents a sample from the underlying probability density. This choice of representation is an important aspect employed by the NPF. On the neuronal level, there has been some support that neurons might indeed represent uncertainty about a stimulus in terms of samples. For instance, it has been shown that inter-trial variability of neuronal responses declines upon stimulus onset^8^, and that this can be related to a decrease in perceptual uncertainty^9^. Moreover, it has been found that spontaneous neuronal activities relate to prior expectations about a stimulus in visual cortex^30^. Since our filtering algorithm is based on unweighted samples, our findings are in line with the advantages of the sampling-based representation outlined by Fiser *et al*.^31^: it can represent any distribution without the need for a parametric form, it avoids the COD and it is well-suited for learning. Filtering approaches implementing Markov-chain Monte Carlo (MCMC) algorithms have received some attention lately^11,12,43,44^, but since they rely on a discrete state space and assume a different coding scheme than the one suggested in Fiser *et al*.^31^, the advantages listed there do not necessarily emerge from these models.

As a filtering algorithm, the NPF is comparable to existing sample-based filtering approaches. Our ansatz may be seen as a particle filter where all particles carry the same weight and which, therefore, avoids numerical pitfalls such as weight degeneracy. This problem is notorious in standard MCMC particle filters^20^ and becomes even more severe as the number of hidden dimensions grows. The COD, i.e. the exponential growth of approximation error with the dimension of the underlying model, is an inevitable nuisance in standard MCMC approaches. Weight degeneracy can be slowed down by particle resampling or by using a more refined propagator for the particles (like the ‘optimal importance function’^20^). However, neither solution is able to mitigate weight decay in general. There exist more elaborate approaches to particle filtering (see eg. refs^34,45,46^) which rely on spatial localization and exhibit a sub-exponential scaling in particles with growing dimensions. Currently, there is no proposed implementation of such a filtering strategy, or even for standard weighted particle methods, in a neural architecture. Since the NPF does not rely on importance weights in the first place, it does not suffer from these numerical pitfalls and their related implementational issues. The COD seems to be avoided by the fact that the observations directly enter the particle trajectories instead of leading to an increasingly fast weight decay (compare ref.^47^), but of course the numerical assessment presented here does not provide a general proof that the NPF avoids the COD in general. For this, further analytical investigations are needed, but go beyond the scope of this article.

In the literature, there have been other approaches for particle filtering without importance weights, derived rigorously from mathematical filtering theory^38,48,49^. One of these approaches is the Ensemble Kalman Filter (EnKF)^49,50^. It is a generalization of the extended Kalman Filter in which copies of the Kalman filter are evolved with a gain that is computed using the empirical variance of the particle samples. As such, for a linear filtering problem, the EnKF outlined in ref.^49^ is equivalent to the NPF with empirical gain factor, but the two algorithms diverge for non-linear problems, where the EnKF has to resort to special techniques in order to remain stable. The FBPF^38^ is based on a similar SDE for the particle trajectories as the one we propose in Eq. (4), i.e. it exhibits the general structure of the hidden SDE as well as novelty-based gain-feedback structure. The FBPF arises as the solution of an optimal control problem, and the optimal feedback gain is the solution of an Euler-Lagrange boundary-value problem (BVP). This BVP cannot be solved in closed form in the multidimensional case, but it can be approximated by a Galerkin method^39^. It is noteworthy that the particle dynamics in the Ensemble Kalman-Bucy Filter (EnKBF) outlined in ref.^50^ are identical to that of the FBPF employing this approximation. The NPF with empirically-adjusting gain *W*_*t*_ resamples the FBPF with Galerkin approximation, up to a slight modification in the novelty term, although the respective approaches to deriving both filters is fundamentally different. Thus far, the main difference between both filters is that a learning framework is provided with the NPF, which allows for learning of model parameters and filter gain factors, whereas the FBPF only accounts for filtering, and not for identification, problems.

We addressed the problem of learning the generative model parameters as well as adjusting the gain matrix *W*_*t*_ by using an online gradient ascent on the logarithm of the likelihood of the observations. For the class of models that we consider, the log likelihood has a very simple representation in terms of the optimal filter. By replacing the optimal filter with the particle estimate of the NPF, the derivation of fully recursive learning rules for the model parameters as well as the gain matrix is straightforward. Presently, we do not have any hard results concerning convergence of the algorithm. For Hidden Markov Models (HMMs) (discrete time and discrete state space), online log-likelihood approaches (but without particles) exist^51,52^ and have recently been shown to converge under relatively mild assumptions^53^. Similar online ML approaches with weighted particle filters (in discrete time) are possible^21^, but are negatively affected by particle degeneracy due to weight decay, and it is an open - yet interesting - question how these results would translate to a weight-free particle method. For HMMs, an online expectation maximization (online EM), which is based on the well-known EM algorithm for offline learning, has been proposed as an alternative to online gradient methods^54,55^. However, at present there are only partial convergence results, and the generalization of online EM to continuous-time models is not fully established. If such a generalization is possible in the future, it will be interesting to see whether the locality problems of online gradient ascent can be resolved.

The question how filtering can be performed by the brain has certainly been addressed before. In particular, filtering algorithms based on linear generative models have been subject to extensive research and mainly study how the analytical solution to this problem, the Kalman filter, can be implemented with neurons^13,14,29,56^. However, the posterior resulting from a Kalman filter is always Gaussian, which is highly restrictive and does not properly reflect activity distributions observed in neurons (compare for instance the observation that neuronal activity is sparse^57^). A more general approach, that does not only suit a nonlinear generative model, but also includes parameter learning is outlined in ref.^58^. It relies on sample paths generated by a biologically plausible neuronal dynamics, which are weighted according to the log likelihood ratio for diffusion processes, and parameters are learned with Expectation Maximization. The important difference to our approach is that both inference and learning are done with respect to the whole path, whereas the NPF is an online algorithm. Another related approach, where neurons are considered Monte Carlo samplers in a hidden Markov model^11,12^, offers both online (nonlinear) filtering and learning in a spiking network. However, the neuronal representation of the hidden state differs considerably to our approach. Indeed, it relies on a discretization of the state-space, and thus we expect this approach to scale unfavorably as the hidden dimension increases.

The neuronal network structure (Fig. 1b) we propose to implement the neuronal dynamics according to Eq. (4) is structurally similar to the one proposed by Rao & Ballard^10^. As in their model, we represent neuronal activities in terms of their instantaneous firing rate, which is an approximation to the spiking nature of biological neurons. In their predictive coding model, a central role is assigned to the predictive error signal, which can be compared to the dynamics of the novelty neurons or novelty signal $d{{\bf{n}}}_{t}^{(k)}$ in each subnetwork. Indeed, recent experimental findings seem to support the existence of neurons that exhibit stimulus-predictive responses^59^. Due to the similarity between predictive coding and the NPF, equations for the neuronal dynamics and for learning the generative weight in the small observation noise limit is similar. However, our model generalizes the one by Rao & Ballard^10^ in the sense that we allow a dynamical prior that is directly reflected in the dynamics of the filtering neurons.

It should be stressed that the NPF as stated in Eq. (4) is an ansatz. This means that other choices that include more biological plausible features are obviously possible. Since the very notion of biological plausibility is not well defined, the point here is not to argue that one specific dynamic is more plausible than another one, but rather to insist on the fact that the NPF does not depend on the importance weights and this provides a great advantage in terms of implementation (being neuronal or silicon-based).

The main limitation of the present framework is that the learning rules are in general not local, a fact mainly due to the so-called filter derivative (see section S 3 in SI). We have seen that for small observation noise, learning rules can be approximated to exhibit Hebbian, and thus local, structure, but we cannot expect the brain to only rely on the observation noise to be small. To overcome nonlocality in the learning rules for larger observation noise, another possibility is to consider a network with only a single particle, which gives us a representation of the posterior in time if the sampler dynamics is fast enough (similarly to an MCMC approach, where the posterior is stationary). We could show that if the decoding weight *W*_*t*_ is learned, the filtering performance of a single-particle NPF is still reasonable (section S 2.3, SI), and might suffice for parameter learning as well. This approach could further be extended by considering *N* independent, i.e. non-communicating filtering networks. Another possibility to overcome nonlocality in the learning rules could be to treat the learning process as an inference and impose a local ansatz for the learning rule. But this goes beyond the scope of this paper.

Irrespective of the limitations and the particular structure of the neuronal network, the two central aspects of our work, namely a sampling-based representation and a filtering algorithm with adaptive gain, result in the following implications: (a) neuronal variability increases with feature uncertainty, (b) neuronal dynamics is driven by the prediction error, i.e. the discrepancy between the predicted observations and the actual observations and (c) the network is robust against neuronal failure.

## Electronic supplementary material

Appendix: Nonlinear Bayesian filtering and learning: a neuronal dynamics for perception
