# Publisher Correction: Nonlinear Bayesian filtering and learning: a neuronal dynamics for perception

**DOI:** 10.1038/s41598-017-17246-9

**Published:** 2017-12-11

**Authors:** Anna Kutschireiter, Simone Carlo Surace, Henning Sprekeler, Jean-Pascal Pfister

**Affiliations:** 1 Institute of Neuroinformatics, University of Zurich/ETH Zurich, Zurich, Switzerland; 2 Neuroscience Center Zurich, University of Zurich/ETH Zurich, Zurich, Switzerland; 3 Department for Electrical Engineering & Computer Science, Technische Universität Berlin, Berlin, Germany; 4 Bernstein Center for Computational Neuroscience, Berlin, Germany

*Scientific Reports* **7**:8722; doi:10.1038/s41598-017-06519-y; Article published online 18 August 2017

The PDF version of this Article was corrupted after publication leading to the incorrect display of equations. This has now been corrected; the HTML version of the paper was correct from the time of publication.

