# Supplementary material for: Nonlinear Bayesian filtering and learning: a neuronal dynamics for perception
Source: Sci Rep. 2017 Aug 18;7:8722. doi: 10.1038/s41598-017-06519-y (PMC5562918; doi:10.1038/s41598-017-06519-y)
Supplement: Supplementary file 1 — Appendix: Nonlinear Bayesian filtering and learning: a neuronal dynamics for perception [file 41598_2017_6519_MOESM1_ESM.pdf]

Appendix: Nonlinear Bayesian filtering and learning: a neuronal dynamics for perception

Anna Kutschireiter, Simone Carlo Surace,
Henning Sprekeler, Jean-Pascal Pfister

S 1 Mathematical background

S 1.1 Stochastic differential equations in a nutshell

For readers who are not familiar with the concepts of Itô stochastic differential equations (SDE), we want to give a very brief overview about how to describe diffusion processes, compute underlying probability distributions and moments from an SDE, and the common discretization scheme that we used to simulate the trajectories.

S 1.1.1 Stochastic differential equations, moments and probability distributions

Consider a vector-valued random variable $\mathbf{x}_t \in \mathbb{R}^n$ that evolves according to an Itô diffusion, i.e. it can be described by the Itô SDE

$$d\mathbf{x}_t = \mathbf{a}(\mathbf{x}_t, t)\, dt + B(\mathbf{x}_t, t) d\mathbf{w}_t. \tag{S-1}$$

Here, $\mathbf{w}_t \in \mathbb{R}^n$ is a vector Brownian motion process with $\langle d\mathbf{w}_t d\mathbf{w}_s^T \rangle = \mathbb{I}^{n\times n}\delta_{ts}dt$, where $\mathbb{I}$ denotes the unit matrix in the corresponding dimension and further $\delta_{ts} = 1$ if $t = s$ and $\delta_{ts} = 0$ otherwise. The deterministic part of Eq. (S-1) is determined by the *drift term* $\mathbf{a}(\mathbf{x}_t, t)dt$ with a vector-valued function $\mathbf{a}(\mathbf{x}_t, t)$, whereas the stochastic part is determined by the *diffusion term* $B(\mathbf{x}_t, t)d\mathbf{w}_t$ with the matrix-valued noise covariance $B(\mathbf{x}_t, t)$.

The process in Eq. (S-1) defines a probability distribution $p(\mathbf{x}_t)$ over the random variable at each time t. In general, the evolution of the probability distribution $p(\mathbf{x}_t)$ is given by the Fokker-Planck equation[1]:

$$dp(\mathbf{x}_t) = \mathcal{L}^\dagger\left[p(\mathbf{x}_t)\right] dt, \qquad \text{with} \tag{S-2}$$

$$\mathcal{L}^\dagger\left[p(\mathbf{x}_t)\right] = -\sum_{i=1}^{n} \frac{\partial}{\partial x_i}\left[a_i(\mathbf{x}_t)p(\mathbf{x}_t)\right] + \frac{1}{2}\sum_{i,j=1}^{n} \frac{\partial^2}{\partial x_i \partial x_j}\left[B_{ij}(\mathbf{x}_t)p(\mathbf{x}_t)\right]. \tag{S-3}$$

where $\mathcal{L}^\dagger$ is called adjoint Fokker-Planck operator [1].

For certain drift and diffusion terms, there exists an analytical solution of Eq. (S-2) for the probability distribution $p(\mathbf{x}_t)$. As an example, let us consider a stochastic process $\mathbf{x}_t$ with drift $a_i(\mathbf{x}) = a_i(x_i)$ and noise covariance $B(\mathbf{x}_t) = \text{diag}(b_i(x_i))$. For this process, the dimensions of the stochastic process are decoupled and the stationary distribution with $dp(\mathbf{x}_t) = 0$ can be computed from Eq. (S-2):

$$p(\mathbf{x}_t) = \prod_{i=1}^{n} p(x_{t,i}) = \prod_{i=1}^{n} \frac{1}{Z_i} \exp\left(\int_{-\infty}^{x_i} \frac{a_i - \frac{1}{2}\frac{\partial}{\partial x''} b_i(x'')|_{x'}}{\frac{1}{2}b_i(x')} dx'\right), \tag{S-4}$$

where Z_i denotes the normalization constant in dimension i.[2]

[1] For the sake of readability, we dropped the explicit t-dependence in the arguments. This, however, does not affect the generality of Eq. (S-2) - (S-6).

[2] Note that depending on $\mathbf{a}(\mathbf{x})$ Eq. (S-4) could for instance be non-normalizable, implying that no stationary distribution exists for this process.

From the Fokker-Planck equation, it is possible to derive the evolution of the expectation of an arbitrary scalar-valued function $\phi(\mathbf{x})$:

$$
\begin{aligned}
d\langle\phi(\mathbf{x_t})\rangle &= \int d\mathbf{x}_t \phi(\mathbf{x}_t)\left(dp(\mathbf{x}_t)\right) \\
&= \left(\int d\mathbf{x}_t \phi(\mathbf{x}_t)\mathcal{L}\left[p(\mathbf{x}_t)\right]\right) dt \\
&= \langle \sum_i a_i(\mathbf{x_t}) \frac{\partial}{\partial x_{t,i}} \phi(\mathbf{x_i}) + \frac{1}{2}\sum_{i,j} B_{i,j}(\mathbf{x}_t) \frac{\partial^2}{\partial x_{t,i}\partial x_{t,j}} \phi(\mathbf{x}_t)\rangle dt \\
&=: \langle \mathcal{L}\left(\phi(\mathbf{x_t})\right)\rangle dt, \qquad \text{(S-5)}
\end{aligned}
$$

where

$$
\mathcal{L}\left[\phi(\mathbf{x}_t)\right] = \sum_{i=1}^{n} a_i(\mathbf{x}_t)\frac{\partial}{\partial x_i}\phi(\mathbf{x}_t) + \frac{1}{2}\sum_{i,j=1}^{n} B_{ij}(\mathbf{x}_t)\frac{\partial^2}{\partial x_i \partial x_j} p(\mathbf{x}_t) \qquad \text{(S-6)}
$$

$\mathcal{L}$ is the Fokker-Planck operator. To sum up, with a given Itô SDE it is possible to set up equations for the evolution of its underlying probability distribution and for any scalar-valued function by determining the Fokker-Planck operator and its adjoint.

S 1.1.2 Euler-Maruyama approximation

For numerical simulation of the SDE in Eq. (S-1) a time-discretization scheme, the so-called the Euler-Maruyama approximation, can be employed [2] to integrate an Itô SDE for small time steps $\delta t = t_{n+1} - t_n$:

$$
\mathbf{x}_{n+1} = \mathbf{x}_n + \mathbf{a}(\mathbf{x}_n, t_n)\delta t + B(\mathbf{x}_n, t_n)\delta \mathbf{w}_n, \qquad \text{(S-7)}
$$

where the increment of the Brownian motion process can be sampled from a Gaussian with zero mean and unit variance, i.e. $\delta\mathbf{w}_n \sim \mathcal{N}(0, \mathbb{I}\delta t)$.

The generative model in our manuscript (cf. Eqs. (S-11) and (S-12)) directly defines the Gaussian transition probabilities $p(\mathbf{x}_t|\mathbf{x}_{t-\delta t})$ of the hidden state variable and the Gaussian emission probabilities $p(\delta\mathbf{y}_t|\mathbf{x}_t)$ for finite time steps δt, which is called Euler-Maruyama approximation, [2]:

$$
\begin{aligned}
p(\mathbf{x}_t|\mathbf{x}_{t-\delta}) &= \mathcal{N}\Big(\mathbf{x}_{t-\delta t} + \mathbf{f}(\mathbf{x}_{t-\delta t})\,\delta t, \Sigma_x\,\delta t\Big), \qquad \text{(S-8)} \\
p(\delta\mathbf{y}_t|\mathbf{x}_t) &= \mathcal{N}\Big(\mathbf{g}(\mathbf{x}_t)\,\delta t, \Sigma_y\,\delta t\Big) \qquad \text{(S-9)}
\end{aligned}
$$

The latter corresponds to the likelihood of the observations $\delta\mathbf{y}_t$. Note that just because the transition and emission probabilities are Gaussian, the marginal probabilities $p(\mathbf{x}_t)$ and $p(\mathbf{y}_t)$ do not necessarily have to be Gaussians. In fact, the shape of the marginal $p(\mathbf{x}_t)$ is determined by the choice of the deterministic drift function $\mathbf{f}(\mathbf{x})$ and the noise covariance Σ_x such that it can be arbitrary. This marginal then constitutes the prior over the real-world variable, which becomes time-invariant if the Fokker-Planck equation has a stationary solution $p(\mathbf{x}_t, t) = p(\mathbf{x})$. The stationary solution of the Fokker-Planck equation for one-dimensional process and state-independent (additive) noise is given by

$$
p(x) \propto \exp\big(-2\frac{\phi(x)}{\sigma_x^2}\big), \qquad \text{(S-10)}
$$

where $\phi(x) = -\int_x f(x')dx'$ is the potential evoked by the deterministic drift.

S 1.2 Nonlinear filtering in a nutshell

In this section, we will outline the formal solution to the filtering problem and introduce to approximate solution, which served as a benchmark in our manuscript: the standard particle filter and the extended Kalman filter.

S 1.2.1 The formal solution to the filtering problem

Recall that the general nonlinear filtering problem is based on the following generative model:

$$d\mathbf{x}_t = \mathbf{f}(\mathbf{x}_t)\,dt + \Sigma_x^{1/2}\,d\mathbf{w}_t, \tag{S-11}$$
$$d\mathbf{y}_t = \mathbf{g}(\mathbf{x})\,dt + \Sigma_y^{1/2}\,d\mathbf{v}_t. \tag{S-12}$$

Solving the filtering problem for the generative model given by Eqs. (S-11) and (S-12) aims at computing the posterior probability of the hidden state $p(\mathbf{x}_t|\mathcal{Y}_t)$, conditioned on the whole sequence of observations up to time t. This problem has already been recognized and tackled by mathematicians in the 60s and 70s of the last century, providing a formal solution for this problem in terms of a (stochastic) partial differential equation for the normalized posterior density, the so-called Kushner equation [3]

$$\begin{aligned} dp(\mathbf{x}_t|Y_t) &= \mathcal{L}^\dagger[p]\,dt + \\ & \Big(d\mathbf{y}_t - \langle \mathbf{g}(\mathbf{x}_t)\rangle_{p(\mathbf{x}_t|Y_t)}\Big)^T \Sigma_y^{-1}\Big(\mathbf{g}(\mathbf{x}_t) - \langle \mathbf{g}(\mathbf{x}_t)\rangle_{p(\mathbf{x}_t|Y_t)}\Big)p(\mathbf{x}_t|Y_t), \end{aligned} \tag{S-13}$$

where $\langle\star\rangle$ denotes the expectation with respect to the posterior probability[3] and $\mathcal{L}^\dagger[\star]$ denotes the adjoint Fokker-Planck operator of the hidden process in Eq. (S-11). The unnormalized posterior density $\rho(\mathbf{x}_t|\mathcal{Y}_t)$ follows the so-called Zakai equation [4]

$$d\rho(\mathbf{x}_t|\mathcal{Y}_t) = \mathcal{L}^\dagger[\rho]\,dt + \mathbf{g}^T\Sigma_y^{-1}d\mathbf{y}_t\,\rho(\mathbf{x}_t|\mathcal{Y}_t). \tag{S-14}$$

Equivalently, the solution to the filtering problem is often given in terms of posterior expectations $\langle\phi(\mathbf{x}_t)\rangle$ of an arbitrary function $\phi(x)$ rather than in terms of the posterior density itself.

$$d\langle\phi\rangle = \langle\mathcal{L}[\phi]\rangle\,dt + \mathrm{cov}\big(\phi,\mathbf{g}^T\big)\Sigma_y^{-1}\big(d\mathbf{y}_t - \langle\mathbf{g}\rangle\,dt\big), \tag{S-15}$$

where $\phi(\mathbf{x})$ is a twice-differentiable, scalar-valued function and $\mathcal{L}$ is the Fokker-Planck operator for the hidden dynamics. For example, $\phi(\mathbf{x}) = \mathbf{x}$ determines the dynamics of the first moment of the posterior distribution, $\boldsymbol{\mu}_t = \langle\mathbf{x}_t\rangle$:

$$d\boldsymbol{\mu}_t = \langle\mathbf{f}\rangle\,dt + \mathrm{cov}\big(\mathbf{x}_t,\mathbf{g}^T\big)\Sigma_y^{-1}\big(d\mathbf{y}_t - \langle\mathbf{g}\rangle\,dt\big). \tag{S-16}$$

The formal solution to the filtering problem is, unfortunately, of little use for practical applications in most cases, because of the so-called closure problem. To illustrate this problem, consider for instance the evolution of the first moment of one component of a one-dimensional hidden variable x for the generative function $g(x) = x$, which we get by plugging $\phi(x) = x$ into Eq. (S-15). The prefactor of the second term in this equation, the covariance $\mathrm{cov}(\phi(x), g(x)) = \mathrm{cov}(x, x)$ will then be a second-order moment. In turn, if we want to compute the evolution of the second moment moment by setting $\phi = x^2$, we end up with a dependence on an even higher-order moment and so forth. This problem has been recognized early on and makes these formal solutions infinite-dimensional. Therefore, in order to solve the filtering problem in a general setting, we need to introduce suitable approximations.

S 1.3 Filtering methods used for comparison

Here, we summarize the algorithms that were used as benchmark in the main manuscript.

S 1.3.1 (Weighted) Particle Filter

Particle filtering is a numerical technique to take samples from the posterior based on sequential importance sampling (see for instance [5] or [6] for review and thorough introduction) in general state-space models. It is easily accessible, because in principle no knowledge of the Fokker-Planck equation or numerical methods for solving partial differential equations is needed. Here, we will briefly outline the algorithm, following [5], and clarify how we used it with our generative model.

[3]For the sake of readability, in the following we will drop the arguments in the functions $\mathbf{f}(\mathbf{x}_t)$, $\mathbf{g}(\mathbf{x}_t)$ and $\phi(\mathbf{x}_t)$, and unless stated explicitly otherwise, expectations $\langle\star\rangle$ are with respect to the posterior $p(\mathbf{x}_t|\mathcal{Y}_t)$.

In general, one considers a state-space model of the form

$$\mathbf{x}_t \sim p(\mathbf{x}_t|\mathbf{x}_{t-1}), \quad \text{(S-17)}$$

$$\mathbf{y}_t \sim p(\mathbf{y}_t|\mathbf{x}_t), \quad \text{(S-18)}$$

where $\mathbf{x}_t$ denotes the hidden process following the prior transition probability $p(\mathbf{x}_t|\mathbf{x}_{t-1})$, and $\mathcal{Y}_t = \{\mathbf{y}_s, s \in \mathbb{N}\}$ is the sequence of observations generated from the emission probability $p(\mathbf{y}_t|\mathbf{x}_t)$ at each time step.

Usually, samples cannot be taken from the filtering distribution $p(\mathbf{x}_t|\mathcal{Y}_t)$ directly. Instead, one takes N samples (commonly referred to as particles) $\mathbf{x}_t^{(i)}$ from a proposal distribution $\pi(\mathbf{x}_t|\mathcal{X}_{t-1}, \mathcal{Y}_t)$ conditioned on the whole history of observations $\mathcal{Y}_t$ and all the previous states $\mathcal{X}_{t-1}$. These samples are weighted according to how well they correspond to the observations with a set of weights $w_t^{(i)}$. The posterior is approximated by

$$p(\mathbf{x}_t|\mathcal{Y}_t) \approx \sum_{i=1}^{N} w_t^{(i)} \delta(\mathbf{x}_t - \mathbf{x}_t^{(i)}) \quad \text{(S-19)}$$

$$\text{with} \quad \sum_{i=1}^{N} w_t^{(i)} = 1. \quad \text{(S-20)}$$

In sequential importance sampling, sampling and reweighing is done recursively at each time step

$$\mathbf{x}_t^{(i)} \sim \pi(\mathbf{x}_t|\mathcal{X}_{0:t-1}^{(i)}, \mathcal{Y}_t), \quad \text{(S-21)}$$

$$\tilde{w}_t^{(i)} = \tilde{w}_{t-1}^{(i)} \frac{p(\mathbf{y}_t|\mathbf{x}_t^{(i)})\, p(\mathbf{x}_t^{(i)}|\mathbf{x}_{t-1}^{(i)})}{\pi(\mathbf{x}_t^{(i)}|\mathcal{X}_{0:t-1}^{(i)}, \mathcal{Y}_t)}, \quad \text{(S-22)}$$

$$w_t^{(i)} = \frac{\tilde{w}_t^{(i)}}{\sum_{j=1}^{N} \tilde{w}_t^{(j)}}, \quad \text{(S-23)}$$

where $\tilde{w}_t^{(i)}$ denotes the unnormalized importance weight of particle i at time t.

For our generative model in Eqs. (S-11) and (S-12), choosing the prior transition probability as the proposal distribution, i.e. $\pi(\mathbf{x}_t|\mathcal{X}_{0:t-1}^{(i)}, \mathcal{Y}_t) = p(\mathbf{x}_t|\mathbf{x}_{t-1}^{(i)})$, we find that Eqs. (S-21) and (S-22) are given by the emission and transition probability defined in Eqs. (S-8) and (S-9), respectively:

$$\mathbf{x}_t^{(i)} \sim p(\mathbf{x}_t|\mathbf{x}_{t-1}^{(i)}) = \mathcal{N}\Big(\mathbf{x}_{t-\delta t}^{(i)} + \mathbf{f}(\mathbf{x}_{t-\delta t}^{(i)})\, \delta t, \Sigma_x\, \delta t\Big), \quad \text{(S-24)}$$

$$\tilde{w}_t^{(i)} = \tilde{w}_{t-1}^{(i)} \mathcal{N}\Big(\delta \mathbf{y}_t - \mathbf{g}(\mathbf{x}_t^{(i)})\, \delta t, \Sigma_y\, dt\Big). \quad \text{(S-25)}$$

This method has two disadvantages. The first one is a problem called weight degeneracy: After a finite number of iterations, all but one of the normalized importance weights are very close to zero, and only one particle will make a significant contribution to the posterior.

In our case, this is indeed catastrophic, because it implies that the posterior is approximated by a single independent sample from Eq. (S-11), which does not take into account the observations at all. To avoid this problem, we re-sample the particles in regular intervals with a probability proportional to their weight whenever the effective number of particles,

$$N_{\text{eff}} = \frac{1}{\sum_j (w^{(j)})^2} \quad \text{(S-26)}$$

is smaller than $N/3$ and set their respective weights to $1/N$ accordingly.

The second shortcoming of this method is that it suffers from the curse of dimensionality, that is an exponential growth of computational complexity as a function of the dimension n of the state vector $\mathbf{x}$. To avoid this problem, more elaborate particle filters would have to be used, as is discussed elsewhere (e.g. [7]).

S 1.3.2 Feedback particle filter

In contrast to weighted particle filtering approaches, the Feedback particle filter (FBPF, [8]) is a particle filter that uses equally weighted particles to approximate the posterior, i.e.:

$$p(\mathbf{x}_t|\mathcal{Y}_t) \quad \approx \quad \frac{1}{N}\sum_{i=1}^{N}\delta(\mathbf{x}_t - \mathbf{x}_t^{(i)}). \tag{S-27}$$

Instead, the observations directly enter the particle dynamics, which evolve according to the Itô SDE (1D model):

$$d\mathbf{x}_t^{(i)} \quad = \quad \left(\mathbf{f}(\mathbf{x}_t^{(i)}) + \Omega(\mathbf{x}_t^{(i)}, t)\right) dt \tag{S-28}$$

$$+\Sigma^{1/2}dB_t^{(i)} + K(x_t^{(i)}, t)\left[d\mathbf{y}_t - \frac{1}{2}\left(\mathbf{g}(\mathbf{x}_t^{(i)}) + \langle\mathbf{g}\rangle\right)\right], \tag{S-29}$$

where $B_t^{(i)}$ are uncorrelated vector Brownian motion processes, $K(x_t^{(i)}, t)$ is the gain matrix and $\langle g\rangle = \frac{1}{N}\sum_{i=1}^{N} g(x_t^{(i)})$ denotes the particle estimate of the observation function. The components of the additional vector-valued drift function $\Omega(\mathbf{x}_t^{(i)}, t)$ are given by [9]

$$\Omega_l(x,t) \quad = \quad \frac{1}{2}\sum_{j=1}^{d}\sum_{k=1}^{m} K_{jk}(x,t)\frac{\partial K_{lk}}{\partial x_k}(x,t). \tag{S-30}$$

The gain K is the solution of an Euler-Lagrange boundary value (ELBV) problem that emerges from an optimal control problem. It is chosen such that it minimizes the Kullback-Leibler divergence between the particle distribution and the posterior filtering distribution (conditioned on proper initialization). In general, K cannot be solved for in closed form, and in practical implementations relies on a numerical solution of the ELBV.

In multiple dimensions, naive approaches to the EL-BVP turn out to be computationally expensive. One way to approach this is to approximate the gain K with a Galerkin approximation. In particular, choosing the coordinate functions as basis functions, the so-called constant gain approximation (CG) reads [9, Eq. 20]:

$$K(x,t) \quad = \quad \frac{1}{N}\sum_{i=1}^{N}\mathbf{x}_t^{(i)}\left(\mathbf{g}(\mathbf{x}_t^{(i)}) - \langle\mathbf{g}\rangle\right)^T. \tag{S-31}$$

In this approximation, the gain is constant with respect to the particle positions, i.e. each particle has the same gain, but still changes as a function of time. In this approximation, the additional drift function Ω in Eq. (S-29) is zero.

We use the FBPF with CG as a benchmark in our multidimensional simulations, because as a related unweighted approach, it does not suffer from the COD (cf. [10]).

S 2 Filtering with the Neural Particle Filter

S 2.1 Limits of small and large observation noise

We want to investigate the limits of small and large observation noise in the NPF and show that these limits are consistent with a Bayesian computation. For the general nonlinear case, these cannot be calculated analytically, but it is possible to do so for a linear generative model, which we would like to outline in the following.

S 2.1.1 The KBF in the limits of small and large observation noise

Before we start analysing the limits of the NPF, let us consider the ground-truth solution to the filtering problem once more: the Kushner equation (Eq. S-13. Intuitively, for large observation noise the observations become meaningless and the Kushner equation becomes the Fokker-Planck equation, i.e. the posterior evolves according to the prior. Conversely, the Kushner equation should be guided entirely by the observation term in the limit of no

observation noise, and for an invertible generative function $\mathbf{g}(\mathbf{x})$[4] the solution should become a delta function $p(\mathbf{x}_t|\mathcal{Y}_t) \sim \delta(d\mathbf{y}_t - \mathbf{g}(\mathbf{x}_t)dt)$. It is tempting to say that of course this is the case, as the second term in Eq. (S-13) is governed by Σ_y^{-1}, meaning it should become large for $\Sigma_y \to 0$ and the Fokker-Planck term should be become negligible. However, as $\Sigma_y \to 0$, so do $\mathbf{g} - \langle \mathbf{g}(\mathbf{x}) \rangle$ and $d\mathbf{y} - \langle \mathbf{g}(\mathbf{x}) \rangle dt$ and it is difficult to tell just from looking at the equation how fast these two contributions approach zero as Σ_y is reduced.

Let us therefore consider an illustrative example of a linear, 1-dimensional system[5]:

$$dx = ax\,dt + \sqrt{\Sigma_x} d\omega_t, \tag{S-32}$$
$$dy = bx\,dt + \sqrt{\Sigma_y} d\nu_t. \tag{S-33}$$

The well-known solution of this problem is a Gaussian with mean μ and variance Σ, whose dynamics is given by the Kalman-Bucy filter [11]:

$$d\mu = a\mu\,dt + \frac{\Sigma}{\Sigma_y} b(dy - b\mu\,dt), \tag{S-34}$$
$$d\Sigma = -\frac{b^2}{\Sigma_y}\Sigma^2\,dt + 2a\Sigma\,dt + \Sigma_x\,dt. \tag{S-35}$$

Equation (S-35) is independent of the innovations process and thus has a well-defined fixed point Σ^*:

$$\Sigma^* = \frac{a\Sigma_y}{b^2} + \frac{1}{b^2}\sqrt{\Sigma_y(b^2\Sigma_x + a^2\Sigma_y)}. \tag{S-36}$$

For $\Sigma_y \to 0$, we find that Σ^* approaches zero with $\Sigma^* \propto \sqrt{\Sigma_y}$. Using this relation, we find that the second term in Eq. (S-34) scales with $\sqrt{\Sigma_y}^{-1}$ and thus dominates the dynamics of μ. Effectively, due to this very large prefactor the time scale of this dynamics $\tau \propto \sqrt{\Sigma_y}$ approaches zero, such that $\dot{y}$ can almost be seen as a constant, and μ relaxes towards $\mu = \dot{y}/b$ almost instantaneously. So in the deterministic limit, the solution is indeed $p(x_t|\mathcal{Y}_t) \simeq \delta(dy_t - bx_t\,dt)$.

On the other hand, in the limit of large observation noise, we find $\Sigma^* = -\frac{\Sigma_x}{2a}$, which corresponds to the variance of the prior probability distribution $p(x)$ determined by Eq. (S-52). Because it is independent of Σ_y, the second term in Eq. (S-34) vanishes and the dynamics of the mean are identical to that of the mean of Eq. (S-52). Therefore, in the limit of large observation noise, the posterior distribution is identical to the prior probability distribution $p(x_t|\mathcal{Y}_t) = p(x_t)$.

S 2.1.2 The NPF in the limits of small and large observation noise

For the empirical version of the NPF with $W = b\Sigma\Sigma_y^{-1}$, the analysis works analogous to the Kalman-Bucy case. For our example, we find:

$$d\mu = a\mu\,dt + \frac{\Sigma}{\Sigma_y} b(dy - b\mu\,dt), \tag{S-37}$$
$$d\Sigma = -\frac{2b^2}{\Sigma_y}\Sigma^2\,dt + 2a\Sigma\,dt + \Sigma_x\,dt. \tag{S-38}$$

Note the factor of 2 that shows up in front of the quadratic term in Eq. (S-38), which seriously affects the steady-state variance Σ^*:

$$\Sigma^* = \frac{a\Sigma_y}{2b^2} + \frac{1}{b^2}\sqrt{\Sigma_y(2b^2\Sigma_x + a^2\Sigma_y)}. \tag{S-39}$$

However, it does not affect the solutions in the deterministic and zero-information limit, respectively, nor the proportionality with $\sqrt{\Sigma_y}$ when letting Σ_y go to zero. Thus, the same reasoning as in the previous sections applies and accordingly, the approximated posterior is the same as the real posterior.

For a nonlinear model, we cannot offer a closed-form solution. However, if we expand the nonlinearities f and g around the mean, which is justified for small observation noise, we obtain equations analogous to Eqs. (S-37)-(S-38)[6], which gives us the same scaling with Σ_y in

[4] A fact that we will assume in the following analysis.

[5] The number of dimensions does not affect the scaling with Σ_y, which is why it readily generalizes to more dimensions

[6] For instance, the gain can be approximated via: $W_t = \mathrm{cov}(x, g(x))\Sigma_y^{-1} \approx g'(\mu)\Sigma\Sigma_y^{-1}$

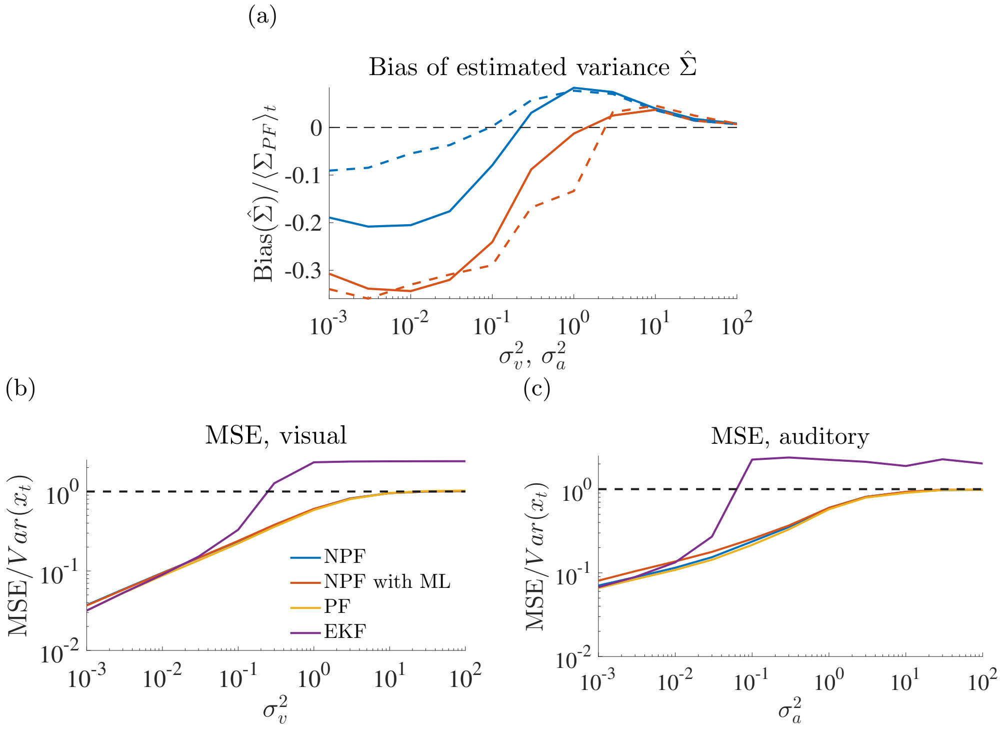

Figure S-1: **Performance and estimation of posterior variance. (a)** The posterior variance $\hat{\Sigma}$ on average exhibits a bias $\text{Bias}(\hat{\Sigma}) = \hat{\Sigma} - \Sigma_{PF}$ when compared to that of the PF. Here, solid (dashed) lines refer to simulations with only the visual (auditory) channel. **(b), (c)** Performance in terms of time-averaged $MSE = \langle(x_t - \hat{x}_t)^2\rangle_t$ for toy model with only visual (b) or auditory (c) cue, i.e. a generative model with Eqs. (15) and (13) or (14), respectively. The gain in the NPF is tuned according to $W_t = \text{cov}(\mathbf{x}_t, \mathbf{g}(\mathbf{x}_t)^T)\Sigma_y^{-1}$ and according to a gradient ascent on the log likelihood ('ML'), respectively For benchmarking, we use a standard PF. The performance of the NPF is nearly indistinguishable to that of the PF. In addition, we compare the performance of an EKF.

the deterministic limit.

S 2.2 Approximation of higher-order moments

Our discussion in the main manuscript assesses the filtering performance of the NPF in terms of it's MSE, which actually measures how close the first moment of the posterior density is to the ground-truth hidden state. However, the we can use the samples to also approximate higher-order moments of the posterior density, or in fact any nonlinear function of hidden state. Even though these approximated moments are not exact (for instance Fig. S-1a for the second moment), the overall posterior shape is still captured to a considerable extent. For some nonlinearities, our proposed model is thus superior to models that are relying on an approximation of just the first two moments of the distribution such as the Extended Kalman Filter (EKF).

For our example from the main manuscript, with a bimodal prior density, the EKF fails to reproduce the features of the (bimodal) posterior, because it is by definition uni-modal. For larger observation noise, the state estimate of the EKF becomes even worse because it evolves to one of the fixed points of f(x) and remains there, irrespective of the real hidden state. This explains why the predictive performance of the EKF in terms of its mean-squared error (MSE) is fairly poor compared to that of the NPF (Fig. S-1b,c).

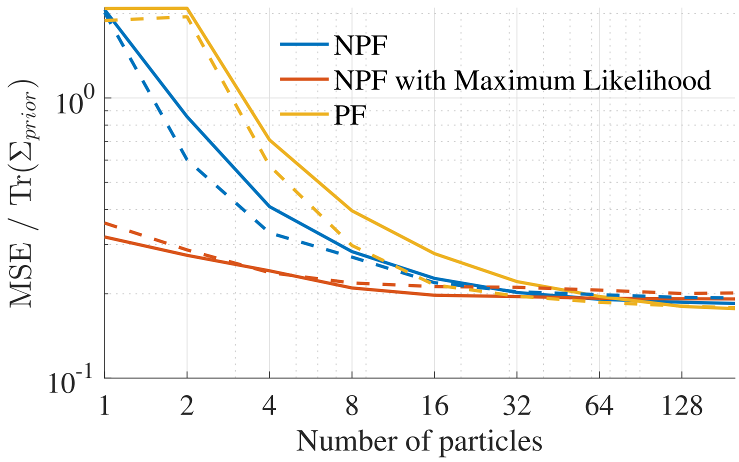

Figure S-2: MSE as a function of the number of particles N in $d = 5$ (solid line) and $d = 1$ (dashed line) hidden dimensions, respectively. Here, the MSE is normalized by the trace of the prior variance to make the error dimension-free. Note that, while the PF and the NPF (with empirical gain) fail completely for a single particle, the NPF with a gain determined by ML has a close-to-optimal performance even for a single particle.

S 2.3 A single-particle estimator

As an interesting side remark we would like to add that the NPF with a gain factor W_t that is learned with ML, can be used as a single-particle estimator. In particular, this filter is able to solve the filtering task with a single particle almost as good as with more particles, as we illustrate in an example in Fig. S-2. By contrast, the NPF with empirically determined gain factor (i.e. relying on a particle distribution to determine the gain) as well as the standard PF (relying on a particle distribution to determine the particle weights) unsurprisingly exhibit an MSE that corresponds to a single independent trajectory of the prior, and thus an MSE of $2 * dim(\mathbf{x})$. Of course, this applies only to the filtering performance as measured by how well the first moment is matched (such as the MSE), and we would like to stress that a single-particle scenario lacks behind in estimation problems that require higher-order moments of the posterior density.

S 3 Parameter learning

S 3.1 The Log Likelihood Function

S 3.1.1 The likelihood function for stochastic processes

Here, we want to outline the derivation of the log likelihood function by Moura & Mitter [12], which we use as a (negative) cost function for parameter learning.

Consider being given a sequence of observations $\mathcal{Y}_t$ that may have been generated by two different diffusion processes, which, in turn, induce two different probability measures $\mathbb{P}$ and $\mathbb{Q}$, respectively. In order to decide which of these processes is more likely to have generated the sequence, a likelihood ratio between these models can be computed, which corresponds to the so-called Radon-Nikodym derivative between the induced probability measures [13, p. 282]:

$$\Lambda(\mathcal{Y}_t) = \frac{d\mathbb{Q}(\mathcal{Y}_t)}{d\mathbb{P}(\mathcal{Y}_t)}. \tag{S-40}$$

Loosely speaking, a large value of $\Lambda(\mathcal{Y}_t)$ provides evidence for the diffusion model inducing $\mathbb{Q}$ and vice versa.

In our model, we have to consider that these observations $\mathcal{Y}_t$ have been generated by a latent process $\mathbf{x}_t$ following Eq. (S-11). Here we introduce the innovations process $\mathbf{n}_t$, following the dynamics

$$d\mathbf{n}_t = d\mathbf{y}_t - \langle \mathbf{g}(\mathbf{x}_t) \rangle_{p_\theta(\mathbf{x}_t|\mathcal{Y}_t)} dt. \tag{S-41}$$

The innovations process is a $\mathcal{Y}_t$-adapted Brownian motion [14, p. 33] under the original measure $\mathbb{Q}_\theta$ (or rather to a family of measures parametrized by θ), which is induced by Eqs. (S-11) and (S-12) with parameters θ and $\Sigma_y = \mathbb{I}$. By rearranging Eq. (S-41), i.e.

$$d\mathbf{y}_t = \langle \mathbf{g}(\mathbf{x}_t) \rangle_{p(\mathbf{x}_t|\mathcal{Y}_t)} dt + d\mathbf{n}_t, \tag{S-42}$$

we thus obtain an Itô SDE for the observations process $\mathbf{y}_t$ under the original measure $\mathbb{Q}_\theta(\mathcal{X}, \mathcal{Y})$, which additionally is completely independent of the latent process $\mathbf{x}_t$, leaving us merely with a measure over the observations $\mathbb{Q}_\theta(\mathcal{Y})$. The objective is now to maximize the likelihood that this process (Eq. S-42) generated the observations with respect to the parameters θ.

In order to determine how likely the observations were generated from the model in Eq. (S-42), we compute the Radon-Nikodym derivative of $\mathbb{Q}_\theta$ with respect to the Wiener measure $\mathbb{P}$, a measure under which $\mathbf{y}_t$ is a Brownian motion process independent of hidden variables as well as parameters.[7] The corresponding Radon-Nikodym derivative can be computed with Girsanov's theorem [14, cf. Eq. 3.18 on p. 52]:

$$\Lambda_\theta(\mathcal{Y}_t) = \frac{d\mathbb{Q}_\theta(\mathcal{Y}_t)}{d\mathbb{P}(\mathcal{Y}_t)} = \mathbb{E}_{\mathbb{P}}\Big[\frac{d\mathbb{Q}_\theta}{d\mathbb{P}}|\mathcal{F}_t^Y\Big] \tag{S-43}$$

$$= \exp\Big(\int_0^t \langle \mathbf{g}(\mathbf{x}_t)\rangle^T d\mathbf{y}_s - \frac{1}{2}\langle \mathbf{g}(\mathbf{x}_s)\rangle^T \langle \mathbf{g}(\mathbf{x}_t)\rangle\, ds\Big). \tag{S-44}$$

Equivalently, instead of maximizing this likelihood function, we consider the logarithm of the likelihood:

$$L_t(\theta) = \int_0^t \langle \mathbf{g}(\mathbf{x}_s)\rangle^T d\mathbf{y}_s - \frac{1}{2}\langle \mathbf{g}(\mathbf{x}_s)\rangle^T \langle \mathbf{g}(\mathbf{x}_s)\rangle\, ds. \tag{S-45}$$

Note that we had to rescale Eq. (S-12) such that it has unit variance. Hence in Eq. (S-45) we have to replace $\mathbf{g}_\theta(\mathbf{x}_t) \to \Sigma_t^{-1/2}\mathbf{g}_\theta$ and $d\mathbf{y}_t \to \Sigma_y^{-1/2} d\mathbf{y}_t$, in order to arrive at (Eq. (9) in main manuscript):

$$L_t^{\text{offline}}(\theta) = \int_0^t \langle \mathbf{g}_\theta(\mathbf{x}_s)\rangle_\theta^T \Sigma_y^{-1}\, d\mathbf{y}_s - \frac{1}{2}\langle \mathbf{g}_\theta(\mathbf{x}_s)\rangle_\theta^T \Sigma_y^{-1} \langle \mathbf{g}_\theta(\mathbf{x}_s)\rangle_\theta\, ds, \tag{S-46}$$

where expectations are taken with respect to the filtering distribution at time s, $p_\theta(\mathbf{x}_s|\mathcal{Y}_s)$. Note that parameter dependence enters via explicit dependence on the parameters θ of the generative function $\mathbf{g}_\theta$ and via implicit dependence on *theta* of the conditional expectation $\langle\cdot\rangle_\theta$.

In a discrete-time approximation, Eq. (S-46) immediately suggests an online maximization scheme. Instead of maximizing the cost function at a time t for the whole observation sequence $\mathcal{Y}_t$, implying we would have to run the filter all over again each time we change the parameters, we just perform a gradient ascent with respect to the parameters θ on the last contribution to the integral, i.e. to

$$L_t^{\text{online}}(\theta) = \langle \mathbf{g}_\theta(\mathbf{x}_t)\rangle_\theta^T \Sigma_y^{-1}\, d\mathbf{y}_t - \frac{1}{2}\langle \mathbf{g}_\theta(\mathbf{x}_t)\rangle_\theta^T \Sigma_y^{-1} \langle \mathbf{g}_\theta(\mathbf{x}_t)\rangle_\theta\, dt, \tag{S-47}$$

where expectations are with respect to the filtering distribution at time t, $p_\theta(\mathbf{x}_t|\mathcal{Y}_t)$.

S 3.1.2 An intuitive derivation of the likelihood ratio

We want to give a less formal justification in discrete time and some intuition and further motivation for the objective function in Eq. (S-44).

The overall objective for parameter learning is the maximization of the likelihood of the observation sequence $\mathcal{Y}_t$. Consider again the innovations process $d\mathbf{n}_t$ (Eq. S-41), which, conditioned on the whole sequence of observations $\mathcal{Y}_t$ up to time t, is a Brownian motion process.

[7] Here, the Wiener measure as a choice of reference is advantageous because the Radon-Nikodym derivative is straightforward to compute.

In discrete time, at each (infinitely small) time step the increment $d\mathbf{y}_t$ is thus distributed according to a Gaussian with mean $\langle \mathbf{g}(\mathbf{x}_t)\rangle_{p(\mathbf{x}_t|\mathcal{Y}_t)}dt$ and variance dt:

$$p(\mathcal{Y}_t) = \prod_{s=0}^{t} p(d\mathbf{y}_s|\mathcal{Y}_{s-1}) \tag{S-48}$$

$$\propto \prod_{s=0}^{t} \exp\left(-\frac{(d\mathbf{y}_s - \langle \mathbf{g}(\mathbf{x}_s)\rangle dt)^2}{2dt}\right) \tag{S-49}$$

$$\Rightarrow \log p(\mathcal{Y}_t) = -\sum_{s=0}^{t} \frac{(d\mathbf{y}_s - \langle \mathbf{g}(\mathbf{x}_s)\rangle dt)^2}{2dt} + \text{const}(dt). \tag{S-50}$$

Considering again the continuous-time limit, the sum in $\log p(\mathcal{Y}_t)$ becomes an integral.

To ensure Eq. (S-50) to be finite, any scaling with $dt^m, m < 1$ is undesirable. However, $d\mathbf{y}_t^2/dt$ is of order dt^0. This term as well as the constant is eliminated by considering instead the logarithm of the likelihood *ratio* between $p(\mathcal{Y}_t)$ and $q(\mathcal{Y}_t)$, where $q(\mathcal{Y}_t)$ is the probability distribution of paths generated by a Brownian motion process with mean 0 and variance dt

$$L_t = \log \frac{p(\mathcal{Y}_t)}{q(\mathcal{Y}_t)} = -\sum_{s=0}^{t} \frac{(d\mathbf{y}_s - \langle \mathbf{g}(\mathbf{x}_s\rangle dt)^2}{2dt} + \sum_{s=0}^{t} \frac{d\mathbf{y}_s^2}{2dt}$$

$$\to \int_0^t \langle \mathbf{g}(\mathbf{x}_s)\rangle^T d\mathbf{y}_s - \frac{1}{2}\langle \mathbf{g}(\mathbf{x}_s)\rangle^T \langle \mathbf{g}(\mathbf{x}_s)\rangle ds. \tag{S-51}$$

Note, that taking this ratio does not affect the maximization of Eq. (S-50), because the denominator is independent of the model parameters as well as the hidden process $\mathbf{x}_t$.

Another, and probably more intuitive, way to look at the objective is the following: Equation (S-50) actually corresponds to a *prediction error*, i.e. the difference between the actual observation $d\mathbf{y}_t$ and its predicted value evaluated from the model parameters and the filtering distribution $\langle \mathbf{g}(\mathbf{x}_t)\rangle_{p(\mathbf{x}_t|\mathcal{Y}_t)}dt$.

S 3.1.3 Bias in the likelihood function for the particle estimate due to approximated posterior

In our model, the fact that we are using *approximations* of the posterior instead of the real posterior $p(\mathbf{x}_t|\mathcal{Y}_t)$, which we don't have access to, in general introduces a bias into the posterior estimation of any function $\langle \phi(\mathbf{x}_t)\rangle$. In the following, we will use the notation $\hat{\phi}_t = \mathbb{E}\big[\phi(\mathbf{x}_t)|\mathcal{Y}_t\big]$ for the *true* posterior estimate (which we in general cannot calculate) and $\langle \phi(x)\rangle_t \approx \frac{1}{N}\sum_{i=1}^{n} \phi(z_t^{(i)})$ for the posterior estimate approximated by the particle positions.

In the general, nonlinear model, the bias $\text{BIAS}(\langle\phi\rangle) = \widehat{\langle\phi\rangle - \phi} = \widehat{\langle\phi\rangle} - \hat{\phi}$ is not analytically accessible. Moreover, this bias not only affects estimation, but also parameter learning, because the likelihood function also depends on posterior estimation and is a random variable even for offline learning[8]. Consequently, the gradient of the log likelihood that is used for parameter learning is biased as well.

Let us illustrate this for a simple, 1-dimensional generative model, which we attempt to solve with an SDE similar to the NPF and for which the bias in the log likelihood can be computed analytically:

$$dx_t = ax_t\, dt + \sqrt{\Sigma_x} d\omega_t, \tag{S-52}$$

$$dy_t = bx_t\, dt + \sqrt{\Sigma_y} d\nu_t. \tag{S-53}$$

The well-known optimal solution of this problem is a Gaussian with mean $\mu_t = \hat{x}_t$ and variance Σ_t, whose dynamics is given by the Kalman-Bucy filter [11].

Assuming stationary of the posterior, there exists a sampler of the form

$$dz_t^{(i)} = \tilde{a} z_t^{(i)}\, dt + \tilde{b}\, dy_t + \tilde{c}\, dw_t, \tag{S-54}$$

[8]Note that for offline learning with the true posterior and for a fixed observation sequence, the log likelihood is deterministic.

which exactly resembles the posterior[9], because of the structural resemblance to the Kalman-Bucy filter (KBF). Consequently, there exists a choice of the gain W_t in the equation of the NPF, such that the first moment of the posterior is matched with the first moment of the KBF. We can compute the bias of the first moment of the single-particle estimator[10]:

$$dz_t^{(i)} = az_t^{(i)}\,dt + W(dy_t - bz_t^{(i)}\,dt) + \sigma_x dw_t, \tag{S-55}$$

$$\Rightarrow d\big(z_t^{(i)} - \hat{z}_t^{(i)}\big) = \big(a - Wb\big)\big(z_t^{(i)} - \hat{z}_t^{(i)}\big)\,dt + \sigma_x dw_t. \tag{S-56}$$

The first moment of the random process described by Eq. (S-56) is equal to the bias of the single-particle estimator, the second moment is equal to the variance of the estimator. Since Eq. (S-56) is an Ornstein-Uhlenbeck process, the stationary solution is given by:

$$\mathrm{BIAS}\big(\langle z^{(i)}\rangle\big) = \lim_{t\to\inf} \widehat{z_t^{(i)} - \hat{z}_t^{(i)}} = 0, \tag{S-57}$$

$$\mathrm{VAR}\big(\langle z^{(i)}\rangle\big) = \lim_{t\to\inf} \widehat{(z_t^{(i)} - \hat{z}_t^{(i)})^2} = \frac{\sigma_x^2}{2(Wb-a)}. \tag{S-58}$$

The bias in the single-particle estimator can be used to compute the bias of the log likelihood L for a limited number of samples. The bias of the log likelihood is given by

$$\mathrm{BIAS}(\tilde{L})_t = \widehat{\tilde{L}}_t - L_t, \tag{S-59}$$

where

$$L_t = \int_0^t b\hat{x}_s\,dy_s - \frac{1}{2}b^2\hat{x}_s^2 ds, \tag{S-60}$$

$$\begin{aligned}\tilde{L}_t &= \int_0^t b\langle x\rangle_s\,dy_s - \frac{1}{2}b^2\langle x\rangle_s^2 ds \\ &= \int_0^t \frac{b}{N}\sum_i z_s^{(i)}\,dy_s - \frac{b^2}{2N^2}\sum_{i,j} z_s^{(i)}z_s^{(j)}ds,\end{aligned} \tag{S-61}$$

are the true log likelihood and the log likelihood estimated from taking N samples, respectively. With Eqs. (S-57) and (S-58) it is straightforward to compute $\widehat{\tilde{L}}_t$:

$$\widehat{\tilde{L}}_t = \int_0^t \frac{b}{N}\sum_i \widehat{z}_s^{(i)}\,dy_s - \frac{b^2}{2N^2}\sum_{i,j}\Big(\frac{\sigma_x^2}{2(Wb-a)}\delta_{ij} + \widehat{z}_t^{(i)}\widehat{z}_t^{(j)}\Big)ds \tag{S-62}$$

$$= L_t - \frac{1}{2}\frac{b^2}{N}\frac{\sigma_x^2}{2(Wb-a)}. \tag{S-63}$$

The second term denotes the bias of the estimated log likelihood. This bias is always negative, i.e. with a finite number of particles we systematically underestimate the true log likelihood, and asymptotically vanishes with increasing number of particles.

This example suggests that gradients for parameter learning should always be taken with respect to the bias-corrected log likelihood. However, this bias cannot be estimated with a nonlinear model, because the true posterior estimated cannot be computed analytically. Thus we have to accept that this bias introduces an error in our parameter estimation that is hard to control. As long as we can be sure that our approximation is asymptotically correct, i.e. in the limit of a large number of particles $N \to \infty$ or an infinitely small binsize $\delta_x \to 0$ in the sampler, respectively, which is correct in the linear case, this bias vanishes asymptotically.

S 3.2 Learning rules

Performing a gradient ascent on Eq. (S-47) with respect to θ gives rise to the online learning rules in Eq. (10) in the main manuscript:

$$\eta_\theta^{-1}d\theta = \Big(\frac{\partial}{\partial\theta}\langle\mathbf{g}(\mathbf{x}_t)\rangle\Big)^T \Sigma_y^{-1}\big(d\mathbf{y}_t - \langle\mathbf{g}(\mathbf{x}_t)\rangle\,dt\big).$$

[9] unpublished work in our group, see also [15]

[10] The single-particle estimator denotes an estimator for the first moment of the posterior created by taking a single sample from the sampling equation.

In our model, we make use of the approximated posterior dynamics in order to derive dynamics of the filter derivative for parameter learning. Equation (10) can be approximated by taking N samples from the NPF equation (4) in order to express the posterior estimates:

$$\langle \mathbf{g}(\mathbf{x}_t) \rangle \approx \frac{1}{N}\sum_{k=1}^{N} \mathbf{g}(\mathbf{z}_t^{(k)}), \tag{S-64}$$

$$\frac{\partial}{\partial\theta}\langle \mathbf{g}(\mathbf{x}_t) \rangle \approx \frac{1}{N}\sum_{k=1}^{N} \left(\frac{\partial \mathbf{g}}{\partial\theta}(\mathbf{z}_t^{(k)}) + G(\mathbf{z}_t^{(k)}) \frac{\partial \mathbf{z}_t^{(k)}}{\partial\theta} \right), \tag{S-65}$$

$$= \frac{1}{N}\sum_{k=1}^{N} \left(\frac{\partial \mathbf{g}}{\partial\theta}(\mathbf{z}_t^{(k)}) + G(\mathbf{z}_t^{(k)}) \boldsymbol{\alpha}_{\theta,t}^{(k)} \right) \tag{S-66}$$

where $G_{ij}(\mathbf{z}^{(k)}) := \frac{\partial g_i}{\partial x_j}(\mathbf{z}^{(k)})$ denotes the Jacobian of the generative function and $\boldsymbol{\alpha}_{\theta,t}^{(k)} = \frac{\partial \mathbf{z}_t^{(k)}}{\partial\theta}$ denotes the filter derivative that takes into account the infinitesimal change in the position of sample $\mathbf{z}^{(k)}$ with respect to the change in parameter value θ.

The single particle filter derivative, $\boldsymbol{\alpha}_{\theta,t}^{(k)}$, cannot be computed directly. However, based on Eq. (4), it is possible to compute its dynamics:

$$d\left(\boldsymbol{\alpha}_{\theta,t}^{(k)}\right) = \frac{\partial}{\partial\theta}(d\mathbf{z}_t^{(k)}). \tag{S-67}$$

Note that every single parameter that is learned has N accompanying filter derivatives of this form. The resulting algorithm is outlined in algorithm S-1.

Algorithm S-1 Parameter learning

1: **procedure** ONLINEML($\theta_{t-\delta t}, \{\boldsymbol{\alpha}_{\theta,t-\delta t}^{(k)}\}_{k=1}^{N}, \{\mathbf{z}_t^{(k)}\}_{k=1}^{N}, \delta\mathbf{y}_t$)
2: **for** $k = 1$ to N **do**
3: Propagate filter derivatives ▷ dynamics for filter derivative are derived from particle dynamics

$$\boldsymbol{\alpha}_{\theta,t}^{(k)} = \boldsymbol{\alpha}_{\theta,t-\delta t}^{(k)} + \frac{\partial}{\partial\theta}(d\mathbf{z}_{t-\delta t}^{(k)}) \tag{S-68}$$

4: **end for**
5: Compute

$$\langle \mathbf{g} \rangle = \frac{1}{N}\sum_{k=1}^{N} \mathbf{g}(\mathbf{z}_t^{(k)}) \tag{S-69}$$

$$\langle \mathbf{g} \rangle_\theta := \frac{\partial \langle \mathbf{g} \rangle}{\partial\theta} = \frac{1}{N}\sum_{k=1}^{N} \left(\frac{\partial \mathbf{g}}{\partial\theta}(\mathbf{z}_t^{(k)}) + G(\mathbf{z}_t^{(k)}) \boldsymbol{\alpha}_{\theta,t}^{(k)} \right) \tag{S-70}$$

6: Update parameter

$$\theta_t = \theta_{t-\delta t} + \eta_\theta \langle \mathbf{g} \rangle_\theta^T \Sigma_y^{-1} (\delta\mathbf{y}_t - \langle \mathbf{g} \rangle \delta t) \tag{S-71}$$

7: **return** $\left(\theta_t, \{\boldsymbol{\alpha}_{\theta,t}^{(k)}\}_{k=1}^{N}\right)$
8: **end procedure**

S 3.2.1 Learning rules for W and J

In this work we are interested in learning the decoding weight matrix W_t and, for a linear observation dynamics $\mathbf{g}(\mathbf{x}) = J\mathbf{x}$, in learning the generative matrix J, respectively. The resulting learning rules for the components of the decoding weight matrix with learning rate η_W are given by

$$dW_{ij} = \eta_W \frac{\partial}{\partial_{W_{ij}}} \langle \mathbf{g}(\mathbf{x}_t) \rangle^T \Sigma_y^{-1} \left(d\mathbf{y}_t - \langle \mathbf{g}(\mathbf{x}_t) \rangle dt \right), \tag{S-72}$$

with filter derivative dynamics

$$d\left(\frac{\partial \mathbf{z}_t^{(k)}}{\partial W_{ij}}\right) = \left(F(\mathbf{z}_t^{(k)}) - WG(\mathbf{z}_t^{(k)})\right)\frac{\partial \mathbf{z}_t^{(k)}}{\partial W_{ij}}dt + \left[d\mathbf{y}_t - \mathbf{g}(\mathbf{z}_t^{(k)})dt\right]_j \mathbf{e}_i, \tag{S-73}$$

where $F_{ij}(\mathbf{z}^{(k)}) = \frac{\partial f_i}{\partial x_j}(\mathbf{z}^{(k)})$ denotes the Jacobian of the nonlinear hidden dynamics and $\mathbf{e}_i$ denotes the unit vector in the i-th direction. This implies that, when we take W_t to be a plastic decoding weight matrix that is learned as observations become available, at least three equations are needed to infer the hidden state at each time step: First, Eq. (4) to evolve the states of the filter neurons, and second, Eqs. (S-72) and (S-73) to update the weights in the filter equation.

Analogously, learning rules for the components of the generative matrix for linear observation dynamics $\mathbf{g}(\mathbf{x}) = J\mathbf{x}$ read

$$dJ_{ij} = \eta_J \left[\left(\frac{\partial\langle \mathbf{x}_t\rangle}{\partial J_{ij}}\right)^T J^T \Sigma_y^{-1}(d\mathbf{y}_t - J\langle \mathbf{x}_t\rangle\, dt) + \left(\Sigma_y^{-1}(d\mathbf{y}_t - J\langle \mathbf{x}_t\rangle\, dt)\langle \mathbf{x}_t\rangle^T\right)_{ij}\right] \tag{S-74}$$

In addition to a term proportional to the filter derivative, the learning rule contains a second term that emerges from an explicit dependence of the likelihood in the generative weight. Filter derivatives are given by

$$d\left(\frac{\partial \mathbf{z}_t^{(k)}}{\partial J_{ij}}\right) = \left(F(\mathbf{z}_t^{(k)}) - WJ\right)\frac{\partial \mathbf{z}_t^{(k)}}{\partial J_{ij}}dt - \hat{x}_{t,j}^{(k)} W \mathbf{e}_i dt. \tag{S-75}$$

S 3.3 Approximation for small observation noise: Hebbian learning

The learning rules we obtain for the decoding weights W_t and the generative weights J are not local, implying that the weights can only be computed when knowing the state of each filter neuron at each time. However, for small observation noise the learning rule for the generative weight J can be approximated by a local learning rule with a Hebbian structure. First, we can neglect the filter derivative, which decays to zero very fast because in this limit, the decoding weight W_t is generally large (cf. Eq. S-75), and thus the first term in Eq. (S-74) vanishes. Second, because in this limit the posterior will approach a δ-distribution around the true hidden state $\mathbf{x}$, as does the approximated posterior, we can approximate the learning rule for J by:

$$\eta_J^{-1} dJ \propto (d\mathbf{y}_t - J\langle \mathbf{x}_t\rangle\, dt)\langle \mathbf{x}_t\rangle^T \approx \langle (d\mathbf{y}_t - J\mathbf{x}_t)\mathbf{x}_t^T\rangle, \tag{S-76}$$

which takes the form of a local Hebbian learning rule.

For numerical illustration, we use our example model from the main manuscript with only a visual cue, i.e. a generative model with Eqs. (15) and (13) Values of the estimator $\hat{J}$ learned with ML, i.e. the learned value of the generative factor, tend to exhibit a slight negative bias, but for an observation noise of up to $\Sigma_y = 0.1$ still stay in a 2%-region below the true generative weight. Hebbian learning leads to an estimator $\hat{J}$ for the generative weight J that is also slightly negatively biased for small observation noise and that becomes less accurate for large observation noise (Fig. S-3a). However, this bias does not seem to affect the filtering performance as measured by the MSE (Fig. S-3).

S 4 Details on numerical experiments

For our simulations, we use a nonlinear hidden dynamics, that was chosen to have a bimodal stationary distribution:

$$dx_t = ax_t(b - x_t^2)\, dt + \sigma_x d\omega_t. \tag{S-77}$$

The parameters $a > 0$ and $b > 0$ can be used to tune the shape of the bimodal distribution, whereby the positions of the two modes is determined by $\pm b$ and a defines how sharply the

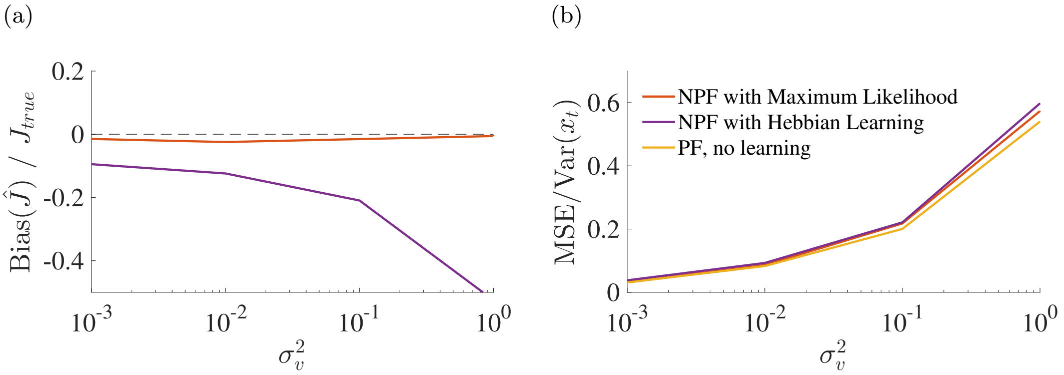

Figure S-3: **Bias of the parameter estimation obtained with a Hebbian learning rule in the limit of small observation noise.** Simulations shown here correspond to the example model with only a visual cue, i.e. a generative model with Eqs. (15) and (13). As benchmark, we use a weighted PF with the true model parameters. **(a)** Bias of estimated generative weight $\hat{J}$ for maximum likelihood and Hebbian learning, respectively. **(b)** The filtering performance is not affected by the bias of $\hat{J}$.

distribution is peaked around the modes. Unless stated otherwise, parameters for the deterministic dynamics were $a = 3$ and $b = 1$, resulting in a bimodal distribution with distinct, but not too sharp peeks at ± 1, such that it is possible for the hidden state to switch from one mode to the other.

In our example simulations in Fig. 3, we employ both linear and sigmoid observation dynamics $g(x)$, thereby simulating multisensory integration with our model (cf. Eqs. 14 and 13). For the plots in Figs. S-1, we use only one sensory modality per subfigure. The observation noise σ_v and σ_a is varied between 10^{-4} and 300.

In the multidimensional simulations in Fig. 5, we use both linear and nonlinear hidden dynamics. We consider the hidden dynamics within each dimension to be independent of the other dimensions, i.e. $f_i(\mathbf{x}) = f(x_i)$, where $f(x) = -x$ for the linear and $f(x) = -3x(1-x^2)$ for the nonlinear hidden dynamics, respectively. $\Sigma_x = \mathbb{I}$ in both cases. The linear generative function is given by $\mathbf{g}(\mathbf{x}) = 2\mathbb{I} \cdot \mathbf{x}$ and $\Sigma_y = \mathbb{I}$.

For the linear model model, the optimal MSE can be computed analytically by a Kalman-Bucy filter in the stationary limit ($\mathrm{MSE}^{\mathrm{opt}} = 0.5 \cdot dim(\mathbf{x})$). For the nonlinear model, the stationary prior is a multimodal distribution with $2^{dim(\mathbf{x})}$ peaks. We determined the optimal performance numerically as the limit of a very large number of particles of the weighted particle method $\mathrm{MSE}^{\mathrm{opt}} \approx 0.42 \cdot dim_x$. The optimal performance is used to compare model performance of the NPF, the FBPF and the PF for a limited number of particles.

Mean-squared errors (MSE) and biases of estimated quantities or parameters $\hat{\theta}$ were computed by

$$MSE = \frac{1}{T}\sum_{t=1}^{T} |\mathbf{x}_t - \langle \mathbf{x} \rangle|^2, \quad \text{(S-78)}$$

$$Bias(\hat{\theta}) = \frac{1}{T}\sum_{t=1}^{T} \hat{\theta}_t - \theta_t, \quad \text{(S-79)}$$

where θ_t denotes the true or benchmark (weighted PF) value. Unless stated otherwise, MSEs are normalized with respect to the trace of the stationary prior variance Σ_{prior} to make performance comparable and, if needed, independent of the number of hidden dimensions.

Other simulation parameters comprise the time step size, which was set to $dt = 0.005$ throughout all simulations. All simulations were run for at least 500'000 time steps, corresponding to 2500 time units. Unless stated otherwise, MSEs and biases were averaged over the last 1000 time units, equaling 200'000 time steps. For the multidimensional simulations, MSEs were determined as an average over the last 5000 time units.

References

[1] Gardiner CW. Handbook of Stochastic Methods. 4th ed. Heidelberg: Springer; 2009.

[2] Kloeden PE, Platen E. Numerical Solution of Stochastic Differential Equations. 1st ed. Berlin, Heidelberg: Springer; 1999.

[3] Kushner H. On the Differential Equations Satisfied by Conditional Probability Densities of Markov Processes, with Applications. Journal of the Society for Industrial & Applied Mathematics, Control. 1962;2(1).

[4] Zakai M. On the optimal filtering of diffusion processes. Zeitschrift für Wahrscheinlichkeitstheorie und verwandte Gebiete. 1969;243.

[5] Doucet A, Godsill S, Andrieu C. On sequential Monte Carlo sampling methods for Bayesian filtering. Statistics and Computing. 2000;10(3):197–208. doi:10.1023/A:1008935410038.

[6] Doucet A, Johansen A. A tutorial on particle filtering and smoothing: Fifteen years later. Handbook of Nonlinear Filtering. 2009;(December 2008):4–6.

[7] Rebeschini P, Van Handel R. Can local particle filters beat the curse of dimensionality? Annals of Applied Probability. 2015;25(5):2809–2866. doi:10.1214/14-AAP1061.

[8] Yang T, Mehta PG, Meyn SP. Feedback particle filter. IEEE Transactions on Automatic Control. 2013;58(10):2465–2480. doi:10.1109/TAC.2013.2258825.

[9] Yang T, Laugesen RS, Mehta PG, Meyn SP. Multivariable feedback particle filter. Automatica. 2016;71:10–23. doi:10.1016/j.automatica.2016.04.019.

[10] Surace SC, Kutschireiter A, Pfister JP. How to avoid the curse of dimensionality: scalability of particle filters with and without importance weights. 2017;(2002):1–16.

[11] Kalman RE, Bucy RS. New Results in Linear Filtering and Prediction Theory. Journal of Basic Engineering. 1961;83(1):95. doi:10.1115/1.3658902.

[12] Moura JMF, Mitter SK. Identification and Filtering: Optimal Recursive Maximum Likelihood Approach; 1986. August.

[13] Klebaner FC. Introduction to Stochastic Calculus with Applications. 2nd ed. Imperial College Press; 2005.

[14] Bain A, Crisan D. Fundamentals of Stochastic Filtering. New York: Springer; 2009. Available from: `http://books.google.com/books?hl=en{&}lr={&}id=hE3KF5Wf6ecC{&}pgis=1`.

[15] Greaves-Tunnell A. An optimization perspective on approximate neural filtering; 2015.
